# Effect of Low Water Availability on Seed Yield and Seed Quality of Basil (*Ocimum basilicum*)

**DOI:** 10.3390/plants12051094

**Published:** 2023-03-01

**Authors:** Iakovos Kalamartzis, George Menexes, Christos Dordas

**Affiliations:** Laboratory of Agronomy, School of Agriculture, Aristotle University of Thessaloniki, 54124 Thessaloniki, Greece

**Keywords:** seed vigor, seed size, germination, yield, cultivar, shoot length, root length, epigenetic

## Abstract

Basil (*Ocimum basilicum* L.) is an aromatic and medicinal plant with important properties and is used as an alternative crop in many countries of the world because of its medicinal, economic, industrial, and nutritional importance. The objective of the present study was to determine the effect of low water availability on seed production and seed quality of five cultivars of basil (Mrs Burns, Cinnamon, Sweet, Red Rubin, and Thai). Irrigation levels and cultivars affected seed yield and thousand seed weight. In addition, plants that were exposed to low water availability produced seeds that germinated in a greater percentage. Additionally, root length was increased as the PEG concentration was increased in the germination solution and was affected by the low water availability of the mother plants. The length of the shoot, the length of the root and the seed vigor could not be used as indicators of low water availability on the mother plants, but these characteristics and especially the seed vigor could be used as indicators of low water availability of the seed. Furthermore, the root length and the seed vigor indicated that there is a possibility of an epigenetic effect of water availability on the seed produced under low water availability, though more work is needed.

## 1. Introduction

Seed production is very important for producing the seeds that are needed for feeding humans and animals and also for sowing the next crop. Seed production is affected by abiotic and biotic factors. One of the most important factors that affect seed production is drought, which is one of the major climatic factors and a crucial constraint for global crop production at all developmental stages from seed germination to ripening [1]. Seed yield losses from low water availability during reproductive stages have been directly linked to a decrease in photosynthetic performance, which leads to a lower synthesis of carbohydrates and also a lower translocation of assimilates to the developing flowers and seeds [2,3]. In addition, low water availability causes weak pollination, higher rates of flower abortion, poor cell division and results in fewer and smaller seeds [4,5]. Therefore, low water availability affects the number and viability of pollen grains and lowers the seed set [6], the number of seeds per plant, the one thousand seed weight (TSW) and the seed yield per area [7,8]. Moreover, low water availability reduces the length of the seed filling period and also speeds up leaf senescence, causing a reduction in seed yield and seed size [9,10,11,12,13,14,15,16,17,18,19,20].

Seed germination is the initial phase of plant growth and is a vital biological process that leads to the development of the seedling which will produce viable offspring. It is regulated by various intrinsic and extrinsic variables [21]. In addition, optimum seed germination is important for successful crop establishment, especially in semi-arid areas [22]. Seed germination begins with water uptake, mobilization and metabolism of stored reserves, and the biosynthesis of proteins and ends with the radicle protrusion from the seed coat [23]. In order for the seedling to maintain its growth, the seed stores resources such as carbohydrates, proteins, and lipids [24]. Moreover, carbohydrates, proteins, and lipids are subjected to hydrolysis by various enzymes and the transport of metabolites takes place when there is adequate water supply [25,26].

In addition, plants encounter multiple environmental stresses and have the ability to withstand abiotic stresses such as heat, drought, salinity, and cold by modifying the morphological, biochemical, physiological, and metabolic changes in plants, directly impacting their growth, development, and productivity. Plants can also adapt quickly to a stressful environment by retaining and sustaining stressful memories, allowing them to withstand repeated stressful situations [27,28,29,30]. These memories are related to epigenetic changes in chromatin, DNA methylation, modifications of histone, chromatin remodeling, phytohormones, and microRNAs [27,28,29,30].

Seed germination of aromatic and medicinal plant species has received special attention in recent years due to the increased demand for these plants in the pharmacological industry, coupled with the need to establish crops for the production of herbs [31,32,33]. High seed quality and seedling establishment are the cornerstones of profitable, efficient, and sustainable crop production, especially for aromatic and medicinal crop plants that are reproduced mainly with seeds such as basil [34]. Basil (*Ocimum basilicum* L.) is an aromatic and medicinal plant that is used extensively because of its important hypoglycemic, anti-inflammation and antispasmodic properties, and its ability to lower blood pressure, reduce fever and body compatibilizer stressors and strengthen the body’s natural activity [35]. In addition, several aromatic and medicinal plants, such as basil, have been introduced in cropping systems as alternative crops in many countries of the world because of their different uses as medicinal, economic, industrial, and nutritional crops [36,37]. The seed size of basil is quite small—the average seed is in the range of 0.28 to 1.8 mg—and special attention should be given to produce a high quality of seeds for use in establishing subsequent crops [38].

There are no studies that show the effect of low water availability on the seed production and seed quality of different basil cultivars under field conditions. The objectives of the present study were (1) to determine the effect of low water availability on seed yield and seed size of five basil cultivars under field conditions and (2) to determine the effect of low water availability on the seed quality—especially on seed germination and seed vigor—of the five cultivars that were exposed to low water availability under field conditions.

## 2. Results

### 2.1. Seed Yield

Seed yield was affected by the irrigation level and the cultivars but not by the year (Table 1). There were significant interactions between the different factors that were studied as seed yield was affected by the interaction of the cultivar with the year and the interaction of the cultivar with the irrigation treatment. Thousand seed weight (TSW) was affected by the cultivar and the interaction of the cultivar with the year and the irrigation level with the year.

**Figure 1:**
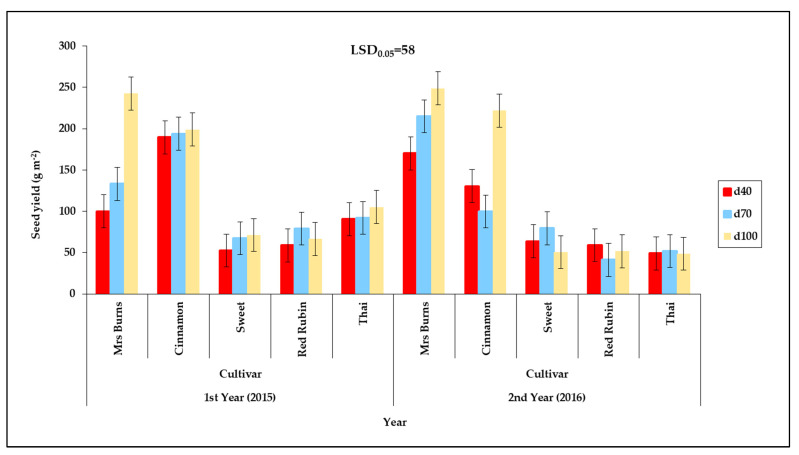
Seed yield of the five basil cultivars (“Mrs Burns”, “Cinnamon”, “Sweet”, “Red Rubin” and “Thai”), at the three irrigation levels, where 40%, 70%, and 100% of the net irrigation requirements (IR_n_) are presented as d_40_, d_70_ and d_100_ respectively, at the two years of experimentation. Data presented are estimated mean values, where LSD is the least significant difference at the 0.05 significance level. Error bars correspond to the estimated common standard error of the mean values. Each mean value has been estimated utilizing *n* = 4 measurements. Figure S1 (see Supplementary material) presents the observed mean values and the corresponding individual standard errors.

The cultivars “Mrs Burns” and “Cinnamon” showed a higher seed yield than the other cultivars (Figure 1). In “Mrs Burns” there was an increase in seed yield as the water availability increased in both years (Figure 1). In addition, “Mrs Burns” and “Cinnamon” showed higher decreases in seed yield with the reduction in water availability, wherein it was increased by 60% between the d_40_ and d_100_ in the cultivar “Mrs Burns” during the first year but the increase was 30% between the d_40_ and d_100_ during the second year.

### 2.2. Thousand Seed Weight (TSW)

**Figure 2:**
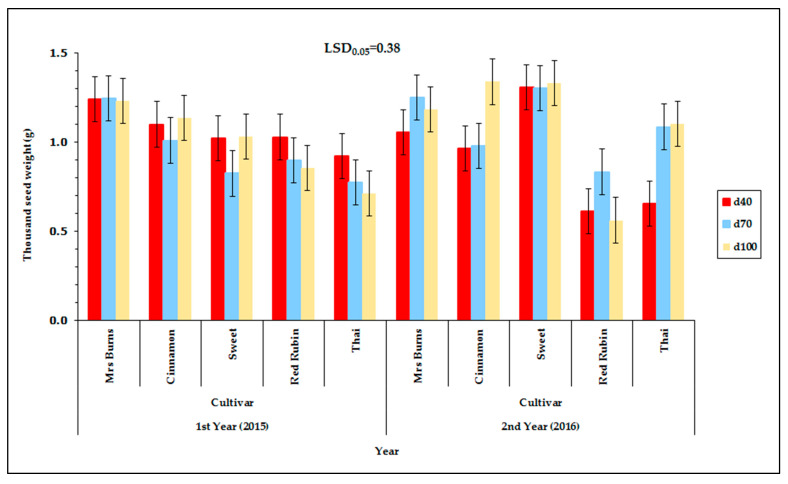
Thousand seed weight (TSW) of the five basil cultivars (“Mrs Burns”, “Cinnamon”, “Sweet”, “Red Rubin”, and “Thai”), at the three levels of irrigation (d_40_, d_70_, and d_100_), at the two years of experimentation. Data presented are estimated mean values, where LSD is the least significant difference at the 0.05 significance level. Error bars correspond to the estimated common standard error of the mean values. Each mean value has been estimated utilizing *n* = 4 measurements. Figure S2 (see Supplementary Material) presents the observed mean values and the corresponding individual standard errors.

Thousand seed weight (TSW) was also affected by the water availability (Table 1). The cultivar that was affected most by water availability was “Thai” and the cultivar “Sweet” proved to be more stable for TSW. During the first year there was no effect of water availability and only the cultivar affected the TSW (Figure 2). During the second year, TSW was reduced for the d_40_ treatment more than for the d_70_ treatment when compared with the d_100_ treatment (Figure 2). There are no other reports that have studied the effect of water availability on TSW. The size of the basil seed is quite small, and it is important to use agricultural practices that maintain or increase the seed size, because larger seeds have higher rates of germination and a higher percentage of survival.

The cultivar “Red Rubin” showed the highest TSW during the first year (0.93 g) while during the second year it was 0.67 g, in contrast the cultivar “Sweet” and “Thai” showed higher TSW during the second year compared with the first year (Figure 2). In particular, “Sweet” showed lower TSW during the first year compared with the second year. A different trend was observed for “Mrs Burns” and “Cinnamon” as the TSW was above one for both years and was 1.24 g for the first year and 1.16 g for the second year for “Cinnamon”. In addition, “Red Rubin” was a late flowering cultivar, and was affected by the dry land conditions. Additionally, the cultivars “Sweet” and “Thai” were affected negatively by the dry land conditions whereas the cultivars “Mrs Burns” and “Cinnamon” were not affected as much by the dry conditions as the cultivar “Red Rubin”, “Sweet”, and “Thai”.

### 2.3. Seed Germination

**Figure 3:**
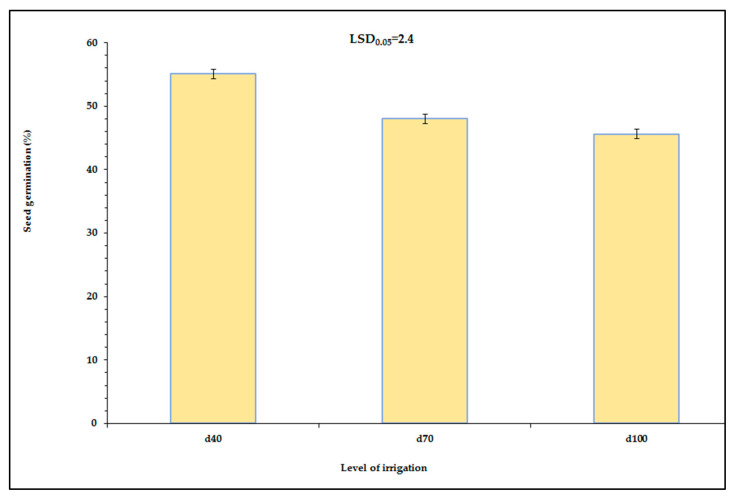
Main effect of irrigation (d_40_, d_70_, and d_100_) on seed germination. Data presented are estimated mean values averaged over all combinations of the levels of the other effects (year, cultivar and PEG concentrations). LSD is the least significant difference at the 0.05 significance level. Error bars correspond to the estimated common standard error of the mean values. Each mean value has been estimated utilizing *n* = 160 measurements. Figure S3 (see Supplementary Material) presents the observed mean values and the corresponding individual standard errors.

Seed germination was affected by the water availability, cultivar, and the PEG concentration of the germination solution (Table 2). In addition, there were significant interactions in seed germination among the different factors that were studied, especially the cultivar × PEG concentration, cultivar × year, PEG concentration × year, and cultivar × PEG concentration × year interactions that were studied (Table 2). Seed germination reduced as the water availability increased, showing 45.6% at d_100_, while at d_70_ it was 48.0%, and at d_40_ it was 55.1%, which means that there was a reduction of up to 17% from the higher to the lower water treatment (Figure 3).

**Figure 4:**
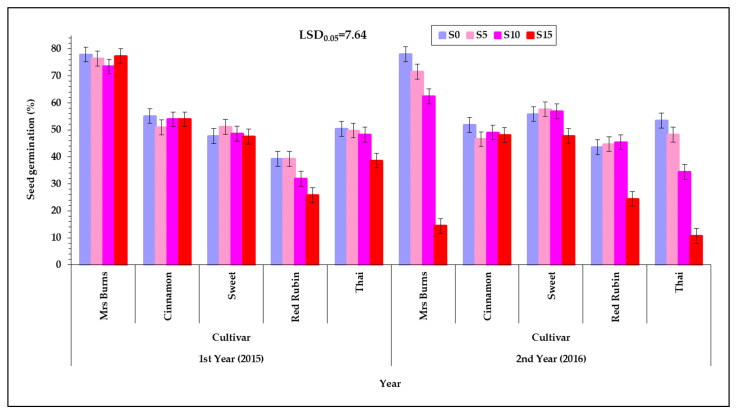
Seed germination of the five basil cultivars (“Mrs Burns”, “Cinnamon”, “Sweet”, “Red Rubin” and “Thai”), at the four PEG concentrations, S_0_ for 0% PEG, S_5_ for 5% PEG, S_10_ for 10% PEG and S_15_ for 15% PEG, at the two years of experimentation. Data presented are estimated mean values averaged over all the three irrigation levels. LSD is the least significant difference at the 0.05 significance level. Error bars correspond to the estimated common standard error of the mean values. Each mean value has been estimated utilizing *n* = 12 measurements. Figure S4 (see Supplementary Material) presents the observed mean values and the corresponding individual standard errors.

There was no interaction in seed germination between the levels of irrigation and the other factors of the experiment (year, cultivar, and PEG concentration) (Table 2). However, there was an interaction among the other factors of the experiments and especially year, cultivar, and PEG concentration. In many cases seed germination was reduced as the PEG concentration of the germination solution was increased (Figure 4). The reduction was higher during the second year and the biggest differences were found between the two concentrations of germination solution S_0_ (55.3%) and S_15_ (38.9%). Figure S4.1 (see Supplementary Material) shows the corresponding raw data used for plotting Figure 4 but in the form of a scatter plot, depicting also the variability among the individual measurements, enhanced with an estimated spline smoothed trend line relative to PEG concentrations. See also Figure S11 in Supplementary Material.

The cultivar “Mrs Burns” showed the highest seed germination in all irrigation treatments, PEG concentration of solution and years at 66.4%, while the cultivar “Red Rubin” showed the lowest values at 41.7%. During the first year, seed germination was not affected in the different PEG concentrations of the solutions with the exception of the cultivars “Red Rubin” and “Thai”, for which the germination was reduced in the highest PEG concentration (Figure 4). During the second year the germination rate was affected in the cultivars “Mrs Burns”, “Red Rubin”, and “Thai” as, in the cultivars “Mrs Burns” and “Thai”, it was reduced as the PEG concentration of the solution was increased, while in the cultivar “Red Rubin” there was a reduction only in the highest concentration of PEG (Figure 4). During the second year in the highest concentration of PEG (15%) the seed germination of the cultivars “Mrs Burns” and “Thai” were reduced at the very low levels of 14.4% and 10.7%, respectively, when compared with the first year.

However, the cultivar “Red Rubin” showed a significant increase in seed germination in the second year compared with the first year in the 10% PEG concentration of the solution (the second year was 45.4%, while the first year was 31.9%). Seed germination of the cultivar “Mrs Burns”, though in most cases high, was drastically reduced at the highest concentration of PEG concentration of the solution. Additionally, seed germination of “Thai” was drastically reduced at the higher concentration of PEG solution. Seed germination of “Sweet” and “Cinnamon” (although lower than “Mrs Burns”) at all concentrations of PEG solutions in both years remained unchanged (differences were not statistically significant). This fact could be considered an important advantage (significant resistance to reduced water availability) on the part of the cultivars “Sweet” and “Cinnamon” and the stability of their seed germination may be of great interest to be confirmed in future field experiments. Additionally, “Red Rubin” showed a relative stability of seed germination but at the highest concentration of PEG (S_15_) showed a statistically significant decrease (39.5% reduction of seed germination) compared with the control.

### 2.4. Effect of the PEG Concentrations on the Length of Shoot of the Seedlings

**Figure 5:**
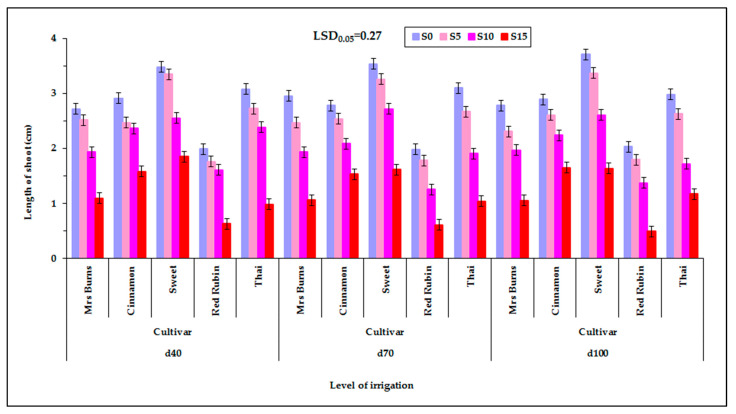
Shoot length of the seedling of the five basil cultivars (“Mrs Burns”, “Cinnamon”, “Sweet”, “Red Rubin” and “Thai”), at the three levels of irrigation (d_40_, d_70_ and d_100_) and at the four PEG concentrations, S_0_ for 0% PEG, S_5_ for 5% PEG, S_10_ for 10% PEG and S_15_ for 15% PEG. Data presented are estimated mean values averaged over the two years of experimentation. LSD is the least significant difference at the 0.05 significance level. Error bars correspond to the estimated common standard error of the mean values. Each mean value has been estimated utilizing *n* = 8 measurements. Figure S5 (see Supplementary Material) presents the observed mean values and the corresponding individual standard errors.

The shoot length of seedlings was affected by cultivar, PEG concentration of the solution and year. Significant interactions were observed between the factors that were studied (Table 2). Shoot length was affected by the interaction of cultivar with PEG concentration, cultivar with year and PEG concentration with year. Additionally, the length of the shoot was affected by the interaction of irrigation level with cultivar and of PEG solution concentration and cultivar with PEG solution concentration and year, which was expected from the existence of the CxS and SxY interactions. Among the levels of irrigation of the mother plants, while statistically significant differences in seedling shoot length were detected, these were not easily detectable (Figure 5). It was found that, at the lowest level of irrigation of the mother plants (d_40_), the characteristic presented in some cases mostly had higher values at S_10_ and S_15_ PEG concentrations versus intermediate irrigation (d_70_) and maximum irrigation level (d_100_). Figure S5.1 (see Supplementary Material) shows the corresponding raw data used for plotting Figure 5 but in the form of a scatter plot, also depicting the variability among the individual measurements, enhanced with an estimated spline smoothed trend line relative to PEG concentrations. See also Figure S12 in Supplementary Material.

**Figure 6:**
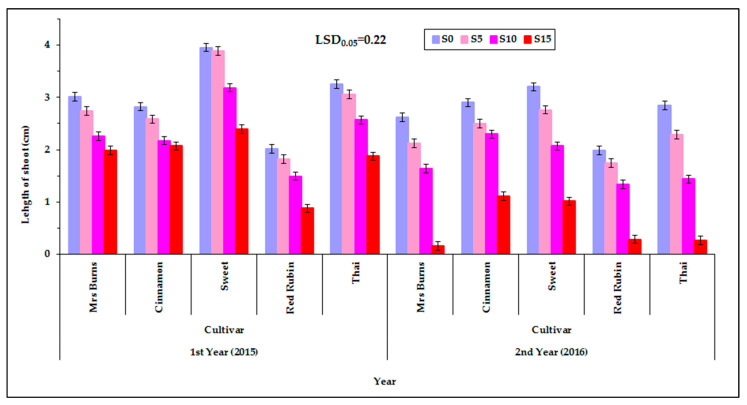
Shoot length of the seedling of the five basil cultivars (“Mrs Burns”, “Cinnamon”, “Sweet”, “Red Rubin” and “Thai”), at the four PEG concentrations, S_0_ for 0% PEG, S_5_ for 5% PEG, S_10_ for 10% PEG and S_15_ for 15% PEG, at the two years of experimentation. Data presented are estimated mean values averaged over the three irrigation levels. LSD is the least significant difference at the 0.05 significance level. Error bars correspond to the estimated common standard error of the mean values. Each mean value has been estimated utilizing *n* = 12 measurements. Figure S6 (see Supplementary Material) presents the observed mean values and the corresponding individual standard errors.

More specifically, at the first year the length of the shoot of the seedling was not different among the solutions of S_0_, S_5_, and S_10_, but it was different between the solutions of S_0_ with S_15_ as well as S_5_ with S_15_ (Figure 6). In these cases, the length of the shoot of the seedling decreased with increasing PEG concentration. At the second year the length of the shoot of the seedlings were not different between the PEG solutions of S_0_ to S_5_ and S_5_ to S_10_ but were different between the solutions of S_0_ to S_10_ (1 cm), S_0_ to S_15_ (2.2 cm), S_5_ to S_15_ (1.7 cm), and S_10_ to S_15_ (1.2 cm). Additionally, in these cases the length of the shoot was decreased with increasing PEG concentration. Therefore, at a moderate S_10_ and high S_15_ concentration of PEG in the germinating solution, the growth of the shoot was limited. Between the control S_0_ and the lower solution concentration S_5_ the decrease in shoot length in some cases (second year) was obvious, while in the next two PEG concentrations, increasing the concentration from S_10_ to S_15_ the decrease in shoot length in most cases (at both years) was much higher (greater than 1 cm) (Figure 6). Meanwhile, the length of the shoot of the seedlings of “Sweet” and “Cinnamon” at the highest concentration of solution S_15_ in both years remained at a satisfactory level. Figure S6.1 (see Supplementary Material) shows the corresponding raw data used for plotting Figure 6 but in the form of a scatter plot, also depicting the variability among the individual measurements, enhanced with an estimated spline smoothed trend line relative to PEG concentrations. See also Figure S12 in Supplementary Material.

### 2.5. Effect of the PEG Concentrations on the Length of Root of the Seedlings

**Figure 7:**
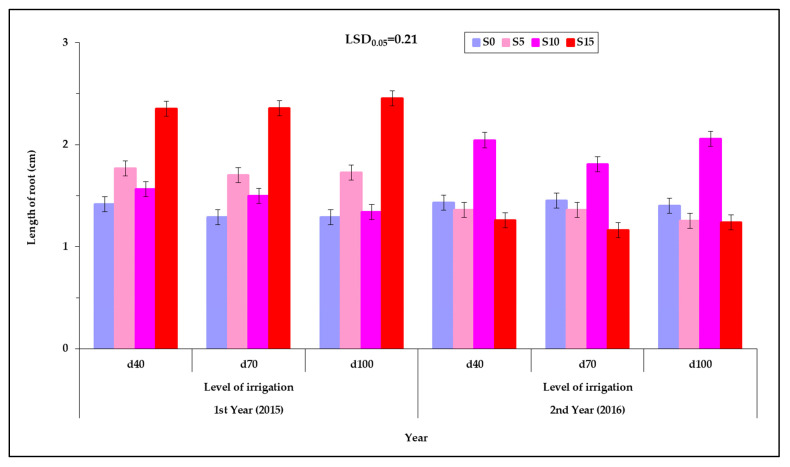
Root length at the three levels of irrigation (d_40_, d_70_ and d_100_), at the four PEG concentrations (S_0_, S_5_, S_10_ and S_15_), at the two years of experimentation. Data presented are estimated mean values averaged over the five cultivars. LSD is the least significant difference at the 0.05 significance level. Error bars correspond to the estimated common standard error of the mean values. Each mean value has been estimated utilizing *n* = 20 measurements. Figure S7 (see Supplementary Material) presents the observed mean values and the corresponding individual standard errors.

The length of the root of the seedling was affected by the cultivar, the PEG concentration of the solution, and the year. Significant interactions were observed between the treatments that were tested (Table 2 and Figure 7). The length of the root was affected by the interaction of the cultivar with the PEG concentration, the cultivar with the year and PEG concentration with the year. Additionally, the length of the root was affected by the three way interaction of the irrigation level with the cultivar and the PEG concentration but also by the interaction of the cultivar with the PEG concentration and by the year (Table 2).

**Figure 8:**
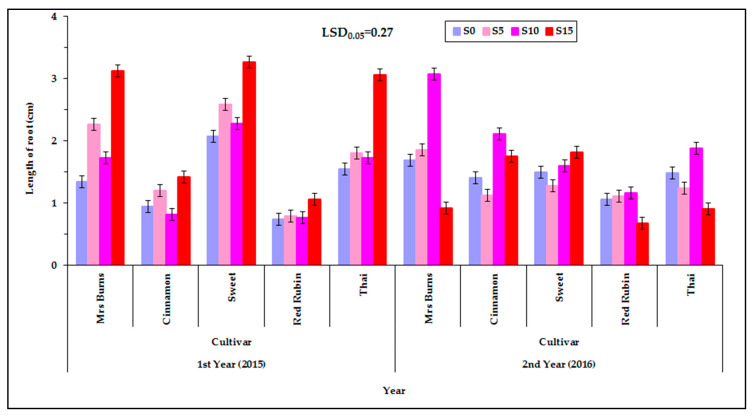
Root length of the five basil cultivars (“Mrs Burns”, “Cinnamon”, “Sweet”, “Red Rubin” and “Thai”), at the four PEG concentrations (S_0_, S_5_, S_10_ and S_15_), at the two years of experimentation. Data presented are estimated mean values averaged over the three irrigation levels. LSD is the least significant difference at the 0.05 significance level. Error bars correspond to the estimated common standard error of the mean values. Each mean value has been estimated utilizing *n* = 12 measurements. Figure S8 (see Supplementary Material) presents the observed mean values and the corresponding individual standard errors.

Among the levels of irrigation of the mother plants, no significant changes in the length of the roots were found, but in most cases the length of the root increased with increasing PEG concentration (Figure 7). At the lowest level of irrigation (d_40_) most cultivars showed a longer root length in the S_10_ treatment (1.8 cm) except for “Sweet” and “Red Rubin” which showed the longest root length in S_15_ treatment (1.8 cm). More specifically, the descending order of classification of cultivars according to the length of the root is as follows: the cultivar “Sweet” for d_40_ and for S_15_ (2.7 cm); “Mrs Burns” for d_100_ and for S_10_ (2.6 cm); “Thai” for d_40_ and for S_10_ (2.1 cm); “Cinnamon” for d_100_ and for S_15_ (1.9 cm); and “Red Rubin” for d_40_ and for S_15_ (1.0 cm) (Figure 8). Figure S7.1 (see Supplementary Material) shows the corresponding raw data used for plotting Figure 7 but in the form of a scatter plot, also depicting the variability among the individual measurements and enhanced with an estimated spline smoothed trend line relative to PEG concentrations. See also Figure S13 in Supplementary Material.

The effect of the PEG concentration on the length of the root was different between the two years 2015 and 2016 (Figure 8). In the first year (2015) the longest root length (2.4 cm) was recorded in the highest PEG concentration (S_15_) while in the year 2016 the longest root length (2.0 cm) was recorded at the concentration of S_10_. It was found that the seed collected in the second year gave seedlings in which the root reached the greatest length in the smallest PEG concentration, while the seeds collected in the first year gave seedlings in which the root reached the greatest length in the greatest concentration of PEG.

In the first year the root lengths were not different between S_0_ and S_10_ and between S_5_ to S_15_ but differed between S_0_ to S_15_ and S_10_ to S_15_. In these cases, the root length increased with increasing PEG concentration of the solution, which was the opposite of that which occurred to the shoot length. At the second year the root length was not different between the solutions except between S_10_ to S_15_. In this case the root length increased with increasing concentration of PEG in a reverse of that which occurred with the length of the shoot (Figure 8). Figure S8.1 (see Supplementary Material) shows the corresponding raw data used for plotting Figure 8 but in the form of a scatter plot, also depicting the variability among the individual measurements and enhanced with an estimated spline smoothed trend line relative to PEG concentrations. See also Figure S13 in Supplementary Material.

### 2.6. Effect of the Factors Studied on the Vigor of the Seed

**Figure 9:**
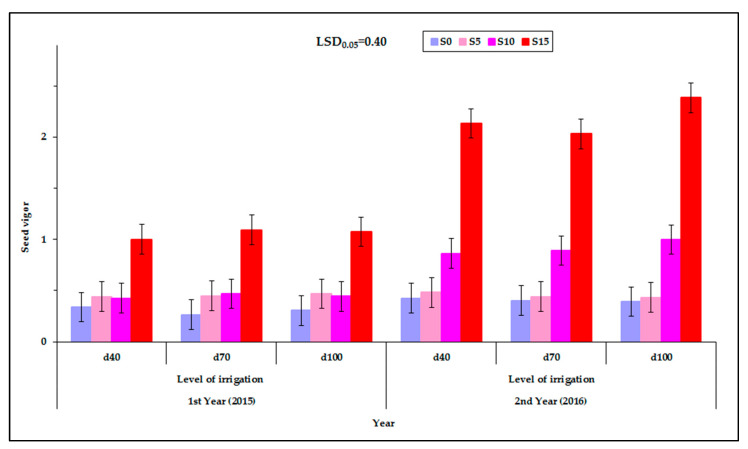
Seed vigor at the three levels of irrigation (d_40_, d_70_, and d_100_), at the four PEG concentrations (S_0_, S_5_, S_10_ and d_15_), at the two years of experimentation. Data presented are estimated mean values averaged over the five cultivars. LSD is the least significant difference at the 0.05 significance level. Error bars correspond to the estimated common standard error of the mean values. Each mean value has been estimated utilizing *n* = 20 measurements. Figure S9 (see Supplementary Material) presents the observed mean values and the corresponding individual standard errors.

The seed vigor was affected by the level of irrigation, the cultivar, the PEG concentration of the solution and the year. Significant interactions between the factors studied were observed (Table 2). The seed vigor was affected by the interaction of the cultivar with the PEG concentration of the germination solution, the cultivar with the year and the PEG concentration of the germination solution with the year. It was expected (from the interactions C×S and S×Y) that the characteristic would be influenced by the interaction of cultivar, PEG concentration, and year (Figure 9). Figure S9.1 (see Supplementary Material) shows the corresponding raw data used for plotting Figure 9 but in the form of a scatter plot, also depicting the variability among the individual measurements and enhanced with an estimated spline smoothed trend line relative to PEG concentrations. See also Figure S14 in Supplementary Material.

**Figure 10:**
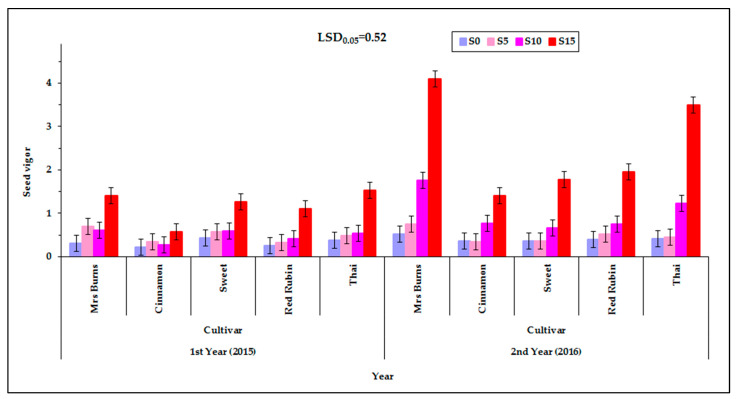
Seed vigor of the five basil cultivars (“Mrs Burns”, “Cinnamon”, “Sweet”, “Red Rubin”, and “Thai”), at the four PEG concentrations (S_0_, S_5_, S_10_, and S_15_) at the two years of experimentation. Data presented are estimated mean values averaged over the three irrigation levels. LSD is the least significant difference at the 0.05 significance level. Error bars correspond to the estimated common standard error of the mean values. Each mean value has been estimated utilizing *n* = 12 measurements. Figure S10 (see Supplementary Material) presents the observed mean values and the corresponding individual standard errors.

Among the levels of irrigation of mother plants, no significant differences in seed vigor were observed. The mean maximum vigor was 4.2 and the mean minimum value was 0.4. It was found that the seed harvested during the first year (2015) had lower vigor than the seed harvested during the second year (2016), at all concentrations of the germination solution (Figure 10). The exception was at S_5_ at which no statistically significant differences were observed between the two years (Figure 10).

For the germination solution, in the majority of cases the highest vigor was recorded at the highest solution concentration S_15_ and in all cases the smallest vigor was found at the control S_0_. An exception was found for “Mrs Burns” which gave the highest vigor (4.2) for the concentration of the S_15_ treatment for the second year (2016) (Figure 9). Additionally, the cultivar “Thai” gave high seed vigor (3.6) in the S_15_ in the second year (2016). During the first year (2015) seed vigor was increased at the PEG concentration of 15% and, similarly, during the second year (2016) the highest seed vigor was also found at S_15_ treatment (Figure 10). Figure S10.1 (see Supplementary Material) shows the corresponding raw data used for plotting Figure 10 but in the form of a scatter plot, also depicting the variability among the individual measurements and enhanced with an estimated spline smoothed trend line relative to PEG concentrations. See also Figure S14 in Supplementary Material.

## 3. Discussion

### 3.1. Seed Yield

Seed yield was affected by the irrigation level and also by the cultivar as the cultivars “Mrs Burns” and “Cinnamon” showed higher seed yield than the others. Seed yield was a characteristic that was affected by the water availability as it was found in other plant species but not in basil [9,13,19,39,40,41]. This reduction in seed yield is probably a result of the lower photosynthesis that basil plants exhibit during periods of reduced water availability. Their leaf area is also reduced, leading to a reduction in produced assimilates and in seed yield [42,43]. Additionally, from this study it is clear that seed yield was affected by cultivar and that there are basil cultivars such as “Mrs Burns” that maintained their growth under low water availability and produce higher seed yield than the other cultivars.

### 3.2. Thousand Seed Weight (TSW)

The size of the seed from the different species of the genus *Ocimum*, of which basil is a part, is quite small, in the range of 0.28 and 1.8 mg per seed [38]. Additionally, the seed size is an important characteristic for seed production, it is important to use agricultural practices that maintain or increase the seed size as larger seeds germinate faster and have a higher percentage of survival under field conditions [38,44]. In addition, there are no other reports that have studied the effect of water availability on the one thousand seed weight (TSW) or on the seed size of basil. Patel et al. [38] reported that the test seed weight was in the range of 0.28 to 1.94 g, which is in the range of the seed weight reported in this study. This may be due to the different cultivars which have different seed sizes. The mean values of TSW that were found in the present study are very similar with the values reported in other studies [45] in which the plants were grown under normal conditions. Basil is reproduced mainly with seeds and the TSW is an important characteristic that shows how well the seed was developed on the mother plant [44]. Moreover, the seed size that is described with the TSW is affected by the rate of photosynthesis and the translocation of carbohydrates and other nutrients from the leaves to the developing seeds [42,43,44].

There was a different trend observed among the cultivars that were tested for “Mrs Burns” and “Cinnamon”, as the TSW was higher than the other cultivars. This is possible because in the first year there were dry conditions and higher temperatures at the end of the season whereas in the second year there were dry conditions at higher temperature during the beginning of the season. In addition, “Red Rubin” is a late flowering cultivar and is affected by dry land conditions more than the other cultivars, as, when drought happens before flowering there is a higher reduction in seed yield during the subsequent flowering and seed development [18,46,47]. Additionally, the cultivars “Sweet” and “Thai” were affected negatively by the dry land conditions whereas the cultivars “Mrs Burns” and “Cinnamon” were not affected by the dry conditions. Moreover, according to the above data, the cultivars can be classified into three groups: the first, wherein the seed size was not affected but the seed yield was affected; a second, wherein the seed size was affected but the seed yield was not affected; and a third, wherein the seed size was affected and the seed yield was also affected.

### 3.3. Seed Germination

Seed germination was increased as the water availability was reduced. This may be due to the epigenetic effect of low water availability on seed development [27,28,29,30,48,49,50,51]. Several biotic and abiotic stresses can cause epigenetic changes in chromatin, DNA methylation, modifications of histone, chromatin remodeling, phytohormones, and microRNAs and these can confer resistance to the next generation [27,28,29,30]. Additionally, it is possible that basil cultivars develop epigenetic mechanisms that are transferred to the next generations when there is limited water supply so that plants are better adapted to dry land conditions [48,49,50,51]. The results from the present study agree with other experiments in other crop species [47,52,53,54,55,56,57,58,59].

In addition, during the second year the weather conditions were drier and with lower rainfall than the first year of the experiment. However, in the second year, compared with the first in the 10% PEG solution concentration, the cultivar “Red Rubin” showed a significant increase in germination percentage (the second year was 45.4% while the first year was 31.9%). The results from the present study agree with other experiments in other crop species [47,52,53,54,55,56,57,58,59].

The concentration of PEG affected seed germination of all cultivars and the germination percentage of the seeds of some cultivars (“Sweet” and “Cinnamon”) at all concentrations of the solutions in both years remained unchanged (differences were not statistically significant). This fact could be considered an important advantage (significant resistance to reduced water availability) of the cultivars “Sweet” and “Cinnamon” and the confirmation under field conditions of the stability of their seed germination may be of great interest. The behavior of the cultivars “Sweet” and “Cinnamon” (germination between 40 and 60%) is close to the germination that has been reported for the cultivars “Dolce Vita” and “Napoletano” (germination rates between 40 and 65%) [59]. Additionally, “Red Rubin” showed a relative stability of the germination percentage of the seed, but at the highest concentration of solution (S_15_) showed a statistically significant decrease (39.5% reduction of the germination percentage compared with the control). Therefore, “Red Rubin” is an interesting cultivar that can be observed under field conditions to confirm their resistance to small and medium reductions of water availability.

### 3.4. Effect of PEG Solutions on the Length of Shoot

It was found that the length of the shoot of the young seedling decreased with increasing concentration of the PEG solution (with increasing osmotic stress) which agrees with other studies [47,52,53,54,55,56,57,58,59]. From the above it was found that there were no epigenetic mechanisms that can act as a tolerance mechanism against osmotic stress. The decrease in the length of the shoot, without exception followed by an increase in the PEG concentration of the solution demonstrates the existence of a reaction to osmotic stress (aqueous stress). This fact may be considered a significant advantage (significant resistance to reduced water availability) of the cultivars “Sweet” and “Cinnamon” and it may be of great interest to confirm this positive reaction to the reduced availability of water in experiments under field conditions.

### 3.5. Effect of PEG Solutions on the Length of Root

The length of the root was increased in most cases as the PEG concentration was increased in the solution. It is possible that this response of the root systems is due to the existence of epigenetic mechanisms, and there is a need for further molecular analysis of the seeds that are produced under low water availability. However, with the observed increase in the length of the root along with the increase in the PEG concentration the idea that the seeds of basil cultivars cope with the lack of water by increasing the length of the root is reinforced [53,59]. This reaction has been recorded in other studies using other plant species [46,60,61,62], and it was found that the length of the main root of the plant increases under low water availability conditions because the plant needs to exploit a larger area of soil for water.

In the present study it was found that the characteristic of the length of the root system is particularly important for the tolerance to low water availability. As a result, the length of the root system, at the stage of the young seedling, can be used as a selection criterion for drought resistance in the early stages of plant growth. However, the results of the different studies (using different plant species) might be due to their differences in the effect of the PEG concentration of the germination solution on the length of the root. There have been studies in which the root behaved like the shoot system by reducing its length by increasing the concentration of the PEG solution (by decreasing the osmotic potential). In particular, Rangel-Fajardo et al. [55] found that in all genotypes the length of the root was reduced as the seeds were exposed to increased PEG concentration. Shtereva et al. [54], Okcu et al. [63], and Bibi et al. [64] found that the length of the root as well as the shoot decreased with increasing PEG concentration. Kalefetogllu Macar et al. [58] have reported that all PEG-containing solutions recorded a decrease in the length of the root system, relative to the control, in all genotypes, except for two genotypes (ILC-3279 and FLIP 87-59C) in which there was no statistically significant difference. However, there have also been studies wherein the length of the root increased with an increase of PEG concentration of the germination solution up to a certain point, as was the case in the present research. Eliege et al. [53], in their experiment, recorded the longest root length in osmotic potential −0.2 and −0.4 MPa. In osmotic potentials of −0.6 and −0.8 MPa the length was shorter and in osmotic potential −1.0 MPa there was no elongation of the root system. According to Ojeda-Silvera et al. [59], in some cultivars of basil (“Lemon” and “Sweet Dani”) the length of the root increased to the solution potential of −0.75 MPa, while in some other cultivars (“Emily” and “Dolly”) the length of the root increased to a solution potential that caused the greatest water shortage (−1.50 MPa), with values that were higher than the values of the other cultivars relative to the control (0 MP_a_), and in the other cultivars the length of the root in this potential (−1.50 MPa) was shorter than in the other solution potentials (0 and −0.75 MPa).

The results of the present study for all cultivars (“Sweet”, “Mrs Burns”, “Thai”, “Cinnamon”, and “Red Rubin”) are consistent with the results of Ojeda-Silvera et al. [59] for the cultivars “Lemon” and “Sweet Dani”, where the length of the root was increased in the intermediate osmotic potential (in the middle osmotic stress) (−0.75 MPa). Similar results were recorded for the cultivars “Emily” and “Dolly”, which maintained the same behavior, of elongation of the root, as an adaptive reaction (as reported by the researchers) to severe osmotic stress (−1.50 MPa) exceeding the control values (0 MPa) [59]. In the remaining cultivars the length of the root at the level of the highest solution concentration decreased more than in the other concentrations [59]. Similar results were recorded in our experiments during the second year (2016) on “Mrs Burns” and “Thai”; however, it was found that in most cases the root length increased with increasing osmotic stress on the seed (increasing PEG concentration of the germination solution) with a few exceptions.

The reaction of the seed to low water availability and in particular to the increase of the root system requires more research. On the one hand, Hamayun et al. [65] examined the growth of soybean seedlings and found that the increase in PEG concentrations led to a significant decrease in the growth of the seedlings, possibly due to a decrease in the enzymatic activity of the hydrolytic enzymes responsible for the hydrolysis of cotyledon reserves, in order to produce the necessary energy required in the early stages of plant growth. On the other hand, there are studies in which it was found that the growth of the root system under conditions of low water availability was greater than that in well-irrigated soil [60,62]. Additionally, others [46,61] have found that the length of the main root of plants increases under stressful water conditions so that it can exploit a larger area of the soil for water. With regard to the present experiment (as well as the experiments of others [53,59]) we can assume that the reaction of the seedling and its individual parts (shoot and root), in most cases, can be interpreted as the ability of the seedling to use the stored nutrients of cotyledons for the benefit of its growth root (more than its shoot) in its attempt to seek water in a substrate where water availability is limited.

### 3.6. Influence of the Factors Studied on the Vigor of the Seed of Basil Cultivars

Seed vigor measured as root/shoot ratio was affected by the factors that were studied (e.g., cultivar, year, irrigation levels, and PEG concentration). The results of this experiment are consistent with the observation that the root/shoot length ratio is increased under osmotic stress conditions, and that this may be due to the greater inhibition of the length of the shoot than the length of root [47]. Additionally, the results of the present study agree with reports about the root length that state that the reaction of the seedling to osmotic stress can be attributed to its ability to use the nutrients that are stored in the cotyledons more towards the growth of the root (than of the shoot) in its effort to find water and to meet its needs for water and survive under stress conditions [46,53,59,61].

## 4. Materials and Methods

### 4.1. Study Site

A field experiment was conducted for two years (2015 and 2016) at the University farm of the Aristotle University of Thessaloniki (40°32′9″ N 22°59′18″ E, 0 m) in Northern Greece. The soil type was a clay loam soil with pH (1:1 H_2_O) 7.77, CaCO_3_ 11.3%, EC (dS m^−1^) 1.07, and organic matter 12.40 g kg^−1^. The soil physical properties were as described previously with a bulk density (Mg m^−3^) 1.3, field capacity (at 10 kPa, m^3^ m^−3^) 0.373, and wilting point (at 1500 kPa, m^3^ m^−3^) 0.132 [42,43]. The previous crop was durum wheat (*Triticum turgidum* subsp. *durum* L.). The cultivation area was prepared for seeding by plowing, harrowing and use of a cultivator. Nitrogen and P fertilizers were applied at the rates of 100 and 50 kg ha^−1^, respectively before planting. Hand weeding and tilling were used for weed control. The weather data were recorded during the experiment, in particular the rainfall, temperature, relative humidity, wind speed, and solar radiation (Table 3), which were recorded daily.

### 4.2. Plant Cultivars Used in the Study

**Figure 11:**
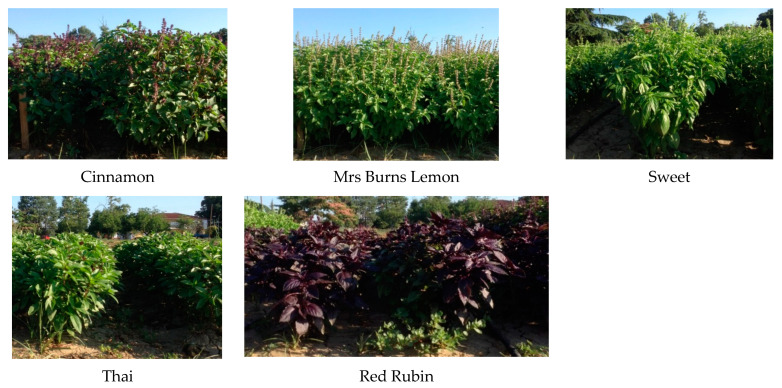
Photographs of the five cultivars that were used in this study.

Five different basil cultivars with differences in agronomic characteristics and also in essential oil content were used in this study. The cultivars that were used have been described previously in Kalamartzis et al. [42,43]. Cinnamon is an early flowering cultivar with a distinctive cinnamon scent. Mrs Burns Lemon is an early flowering cultivar with a distinctive lemon scent. Sweet is a medium flowering cultivar, a new hybrid of the Genovese type cultivar, with a more pointed leaf, is vigorous with good uniformity, slow to flower, and has broad spectrum tolerance to Fusarium wilt. Thai is a late flowering cultivar with a mild anise or liquorice flavor and with attractive purple stems and dark green leaves that are tinged purple. Finally Red Rubin is a late flowering cultivar with a good red for cut leaf or pot production (Figure 11).

### 4.3. Crop Management and Experimental Design

The experiments described in this study for seed yield and one thousand seed weight (TSW) were set up on a randomized complete block design (RCBD) in a split plot arrangement with four replications (blocks). The main plots were the irrigation levels, and the sub-plots were the cultivars. Each block was divided into three strips corresponding to the three irrigation levels and within each strip the five cultivars were randomized. Every plot had five rows, the length of each row was 5 m, and the rows were 50 cm apart, the total size of each plot was 12.5 m^2^ with 64 plants per plot. Seeds were sown in a mixture of peat and perlite (9:1) on the 4th of April 2015 and 19th of March 2016. When the basil seedlings reached 10 cm in plant height, they were hand-planted on 16 May 2015 and on 25 April 2016 at a rate of 8 plants m^−2^.

Briefly, irrigation treatments that were applied were 40%, 70%, and 100% of the net irrigation requirements (IR_n_) and are presented as d_40_, d_70_, and d_100_ respectively. IR_n_ was calculated from the equation:

IR_n_ = ET_c_ − P_e_ − CR + D_p_ + R_off_ (1)

where ET_c_ is the crop evapotranspiration; P_e_ is the effective rainfall, which was taken into account only when it was higher than 4 mm on any day and the entire rainfall was considered as effective rainfall; CR is the capillary rise from the groundwater table; D_p_ is the deep percolation; and R_off_ is the runoff. In this study, the CR, D_p_ and R_off_ were negligible because (a) there is no shallow water table problem in the experimental area, thus CR value was assumed to be zero; (b) D_p_ was not assumed since the amount of irrigation water was equal to the deficit amount in the root zone; and (c) irrigation was performed with drip irrigation and there was no runoff.

Reference evapotranspiration (ET_o_) was calculated with the FAO Penman–Monteith method [66] with the following equation:

ET_o_ = [0.408Δ(R_n_ − G) + γ[900/(T + 273)]u_2_(e_s_ − e_a_)]/[Δ + γ(1 + 0.34u_2_)] (2)

where ET_o_ is the reference evapotranspiration (mm day^−1^), R_n_ is net radiation at the crop surface (MJ m^−2^ day^−1^), G is soil heat flux density (MJ m^−2^ day^−1^), T is mean daily air temperature at 2 m height (°C), u_2_ is wind speed at 2 m height (m s^−1^), e_s_ is saturation vapor pressure (kPa), e_a_ is actual vapor pressure (kP_a_), e_s_ − e_a_ is the saturation vapor pressure deficit (kPa), Δ is the slope vapor pressure curve (kP_a_ °C^−1^), γ is the psychrometric constant (kPa °C^−1^).

Crop evapotranspiration (ET_c_) was calculated with the following equation:

ET_c_ = k_c_ × ET_ο_ (3)

where k_c_ is the crop coefficient.

The following values of crop coefficient (k_c_) were used: for the beginning of flowering 0.9, for full bloom 1.1 and for the end of flowering 1.0 [67].

Soil moisture was kept at 70% of field capacity which is considered adequate for plant growth in all growth stages at full irrigation (d_100_). The differentiation of irrigation levels started when the plants were at the vegetative stage, 40 days after transplantation and 30 days before anthesis. After transplantation, 30 mm of irrigation water was applied in order to promote the establishment of the newly transplanted plants. The water was applied with a drip irrigation system, after transplanting with the drippers spaced at 50 cm intervals the water supply of the drippers was 4 L h^−1^. The drip irrigation lines were placed every other row. The same irrigation system was extensively used in other experiments and the same treatments [42,43,68].

### 4.4. Seed Yield

The seeds were harvested from an area of 2 m^2^ from each plot and placed to dry for a week. When the seeds were dry the biomass was weighted and then the seeds were obtained with an LD 350 laboratory thresher (Wintersteiger AG, Austria) in the first week of September in both years. The seeds were cleaned and weighted to determine the seed yield.

### 4.5. One Thousand Seed Weight

One thousand seeds were counted and weighted to determine their weight.

### 4.6. Seed Germination

One hundred seeds of each genotype and from each treatment were germinated on filter paper in petri dishes. Osmotic stress was induced using PEG-6000 at four different concentrations: 0, 5, 10, and 15%. The solutions were prepared in distilled water. The experimental design was a randomized complete block design (RCBD) with four replicates (blocks) in a split-split plot arrangement: “irrigation levels” were the main plots, “cultivars” were the sub-plots, and “PEG concentrations” were the sub-sub plots. Seeds were considered as germinated when the radicle reached at least 2 mm in length. Shoot and root length were measured with a ruler and seedling vigor was calculated by diving the root length with the shoot length from 20 seedlings.

### 4.7. Statistical Analysis

Data for seed yield and thousand seed weight were analyzed within the methodological frame of mixed linear models with the analysis of variance (ANOVA) method, according to the model that involves the effects (main and interactions) of three factors: 2 “years” × 3 “irrigation levels” × 5 “cultivars”. The experiment was installed according to the randomized complete block design (RCBD) in a split-split plot arrangement, utilizing data from four blocks per combined treatment. The “years” were considered as the main plots, “irrigation levels” were considered as the sub plots, and “cultivars” were considered as the sub-sub plots [69,70]. A combined over years ANOVA was performed according to the previously described experimental setup. For seed germination, length of shoot, length of root and seed vigor the basic experimental design within each year of experimentation was the RCBD (4 blocks) in a split-split plot arrangement: “irrigation levels” were the main plots, “cultivars” were the sub plots, and “PEG concentrations” were the sub-sub plots. The combined ANOVA over years corresponds statistically to a split-split-split plot analysis, where the levels of the factor “years” are considered as the main plots, the irrigation levels are considered as the sub plots and so on. Pair-wise differences among the mean values of the examined effects (main and interactions) were tested with the protected least significant difference (LSD) criterion. All mean values were estimated from the corresponding linear models. The ANOVA method was used mainly for estimating the correct standard errors of the differences among mean values compared with the LSD criterion that was used to test all interesting comparisons. The significance level of all hypothesis testing procedures was preset at *a* = 0.05 (*p* ≤ 0.05). The IBM SPSS Statistics v.23 software was used for the statistical analyses. One of the authors developed and programmed a special SPSS syntax code to perform the statistical analysis and the testing of the proposed mixed linear model. For all parameters examined (seed yield, thousand seed weight, seed germination, length of shoot, length of root and seed vigor) the main effects (mean and median values) of the corresponding statistical models are presents in Supplementary Tables S1–S7.

## 5. Conclusions

The reduction in irrigation water during the seed development in the mother plants resulted in reduced seed yield and reduced TSW. Additionally, there was an immediate response to the treatment of reduced water availability for the next generation of plants, so that plants that received a limited amount of water produced seeds that germinate to a greater percentage. The length of the shoot, the length of the root and the seed vigor (shoot/root length ratio) could not be used as indicators of low water availability for the mother plants, but these characteristics, and especially the seed vigor, could be used as indicators of low water availability (osmotic stress) for the seed. In addition, the root length indicates that there is a possibility of an epigenetic effect of low water availability of the mother plant on the seed that is produced; however, more work is needed.

## Figures and Tables

**Table 1:** Analysis of variance results (significance of the effects) for testing the effects (main and interactions) of year (Y), irrigation (W), and cultivar (C) on the seed yield and one thousand seed weight (TSW).

Parameters	Year (Y)	Irrigation (W)	Cultivar (C)	C × Y	W × Y	C × W	C × Y × W
Seed yield	NS	*	*	*	NS	*	NS
TSW	NS	NS	*	*	*	NS	NS

* Statistically significant effect at significance level *a* = 0.05 (*p* ≤ 0.05). NS: Non-statistically significant effect (*p* > 0.05).

**Table 2:** Analysis of variance results (significance of the effects) for testing the effects (main and interactions) of year (Y), irrigation (W), PEG concentration of the solution (S) and cultivar (C) on the seed germination, length of shoot, length of root and seed vigor.

Effects	Parameters			
	Seed Germination	Length of Shoot	Length of Root	Seed Vigor
Year (Y)	NS	*	*	NS
Irrigation (W)	*	NS	NS	NS
Cultivar (C)	*	*	*	NS
PEG concentration (S)	*	*	*	NS
W × C	NS	NS	NS	NS
W × S	NS	NS	NS	*
W × Y	NS	NS	NS	NS
C × S	*	*	*	*
C × Y	*	*	*	*
S × Y	*	*	*	*
W × C × S	NS	*	*	*
W × C × Y	NS	NS	NS	NS
W × S × Y	NS	NS	NS	NS
C × S × Y	*	*	*	*
W × C × S × Y	NS	NS	NS	NS

* Statistically significant effect at significance level *a* = 0.05 (*p* ≤ 0.05). NS: Non-statistically significant effect (*p* > 0.05).

**Table 3:** The main weather parameters (Rainfall, maximum (T_max_), minimum (T_min_), and mean (T_mean_) temperature), for the two years. The weather data were recorded with an automatic weather station close to the experimental site.

	Year 2015				Year 2016			
	June	July	August	September	June	July	August	September
T_max_ (°C)	29.8	34.3	33.8	29.2	32.4	34.5	32.8	27.4
T_min_ (°C)	17.1	20.5	20.4	14.6	18.7	21.2	19.2	12.2
T_mean_ (°C)	23.2	27.5	27.1	26.2	25.9	27.8	26.1	26.4
Rainfall (mm)	96.2	8.2	1.1	20	15.2	1.2	0.8	18

## Data Availability

Not available.

## Supplementary Materials

The following supporting information can be downloaded at: https://www.mdpi.com/article/10.3390/plants12051094/s1.
