# Supplementary material for: Effect of Low Water Availability on Seed Yield and Seed Quality of Basil (Ocimum basilicum)
Source: Plants (Basel). 2023 Mar 1;12(5):1094. doi: 10.3390/plants12051094 (PMC10005375; doi:10.3390/plants12051094)
Supplement: Supplementary file 1 [file plants-12-01094-s001.zip › Supplementary Figures.pdf]

Supplementary material: Figures

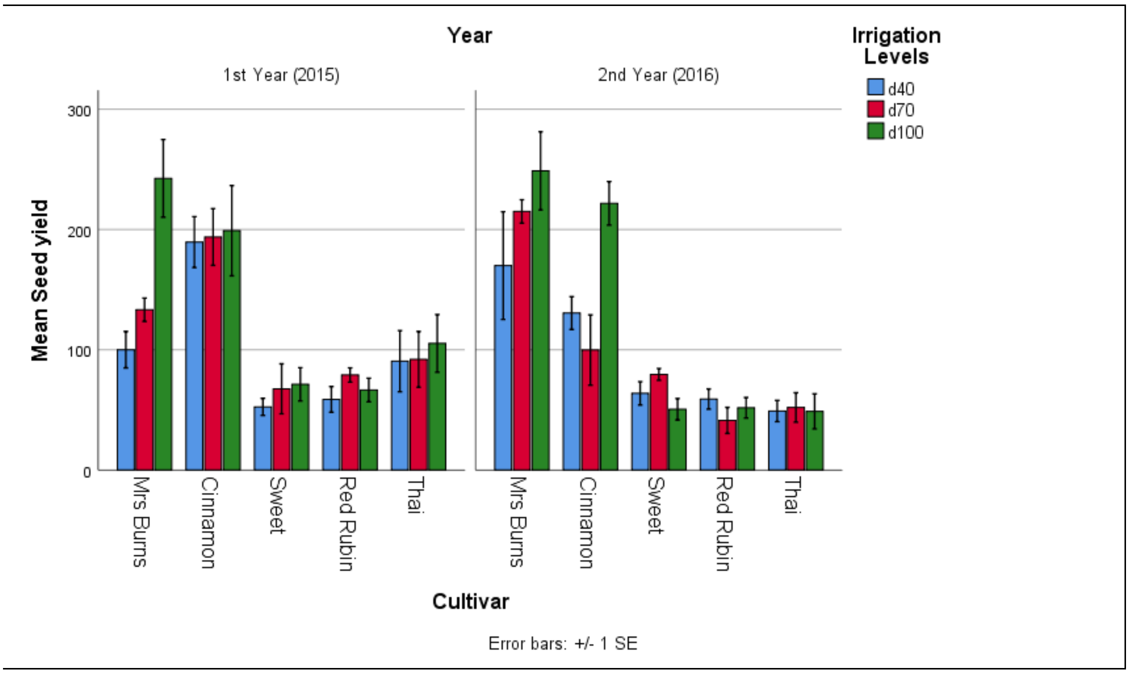

Figure S1. Seed yield of the five basil cultivars ("Mrs Burns", "Cinnamon", "Sweet", "Red Rubin" and "Thai"), at the three irrigation levels where 40%, 70%, and 100% of the net irrigation requirements (IRn) and are presented as d_{40}, d_{70} and d_{100} respectively, at the two years of experimentation. Data presented are the observed mean values. Error bars correspond to the individual standard errors of the mean values. Each mean value has been estimated utilizing *n*=4 measurements.

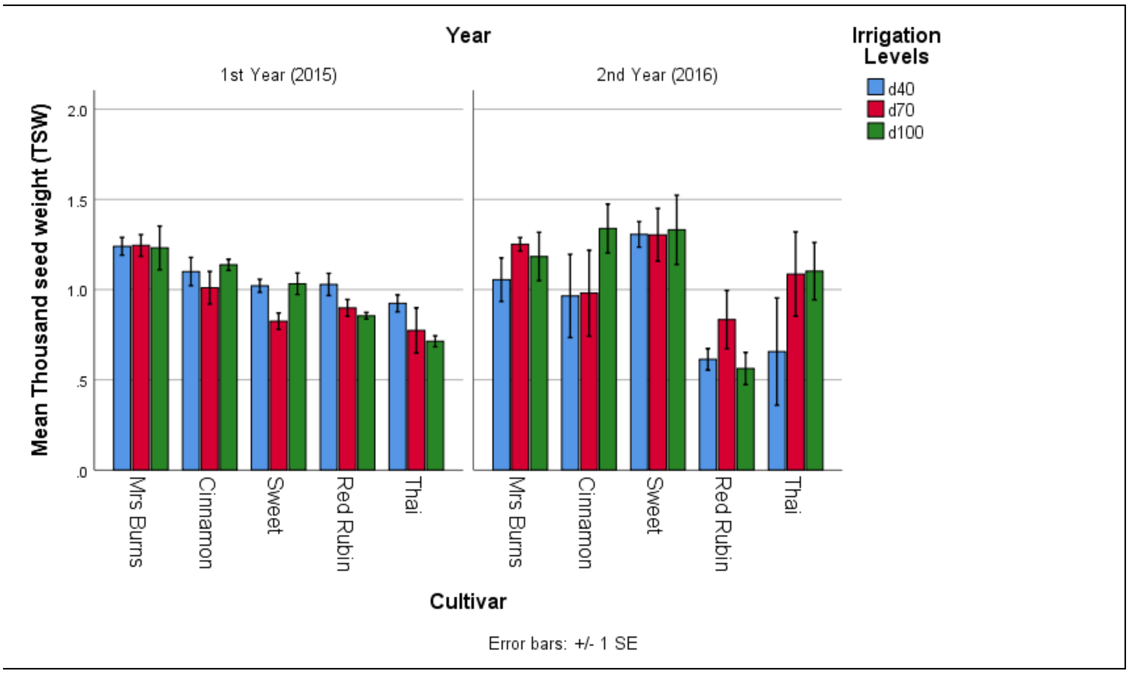

Figure S2. Thousand Seed Weight (TSW) of the five basil cultivars ("Mrs Burns", "Cinnamon", "Sweet", "Red Rubin", and "Thai"), at the three levels of irrigation (d_{40}, d_{70}, and d_{100}), at the two years of experimentation. Data presented are the observed mean values. Error bars correspond to the individual standard errors of the mean values. Each mean value has been estimated utilizing n=4 measurements.

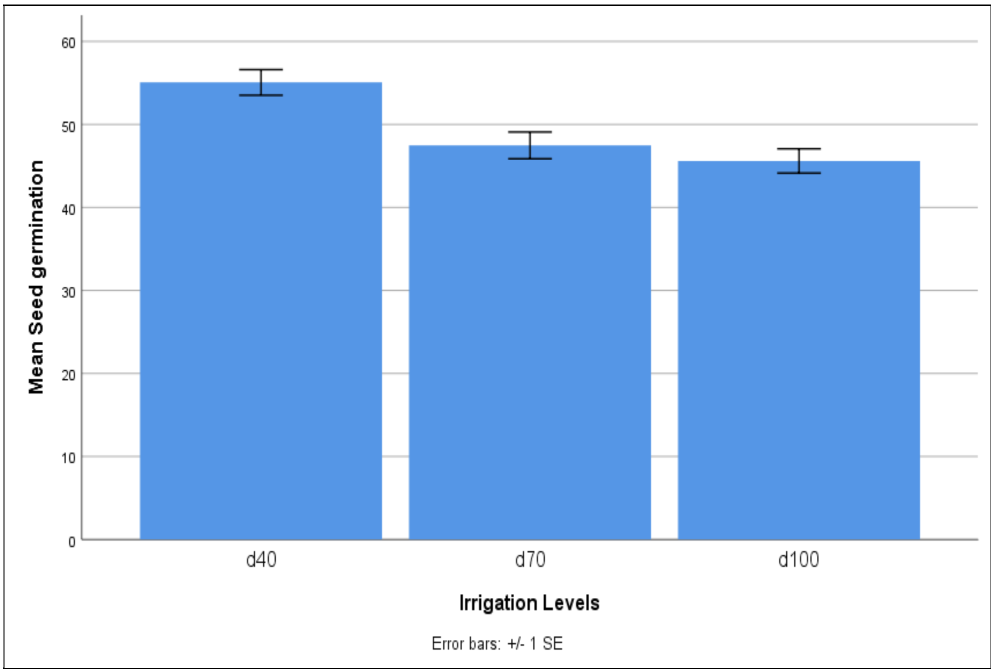

Figure S3. The main effect of irrigation (d_{40}, d_{70}, and d_{100}) on seed germination. Data presented are the observed mean values averaged over all combinations of the levels of the other effects (Year, Cultivar and PEG concentrations). Error bars correspond to the individual standard errors of the mean values. Each mean value has been estimated utilizing n=160 measurements.

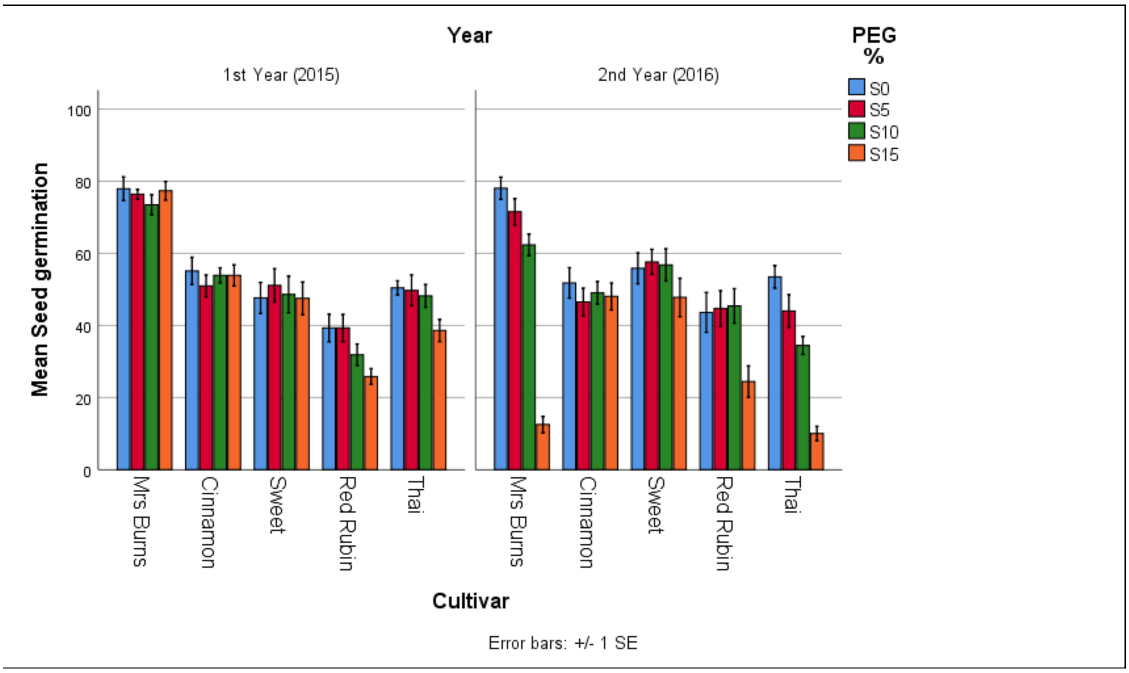

Figure S4. Seed germination of the five basil cultivars ("Mrs Burns", "Cinnamon", "Sweet", "Red Rubin" and "Thai"), at the four concentrations of PEG, S_0 for 0% PEG, S_5 for 5% PEG, S_{10} for 10% PEG and S_{15} for 15% PEG, at the two years of experimentation. Data presented are the observed mean values averaged over all the three irrigation levels. Error bars correspond to the individual standard errors of the mean values. Each mean value has been estimated utilizing n=12 measurements.

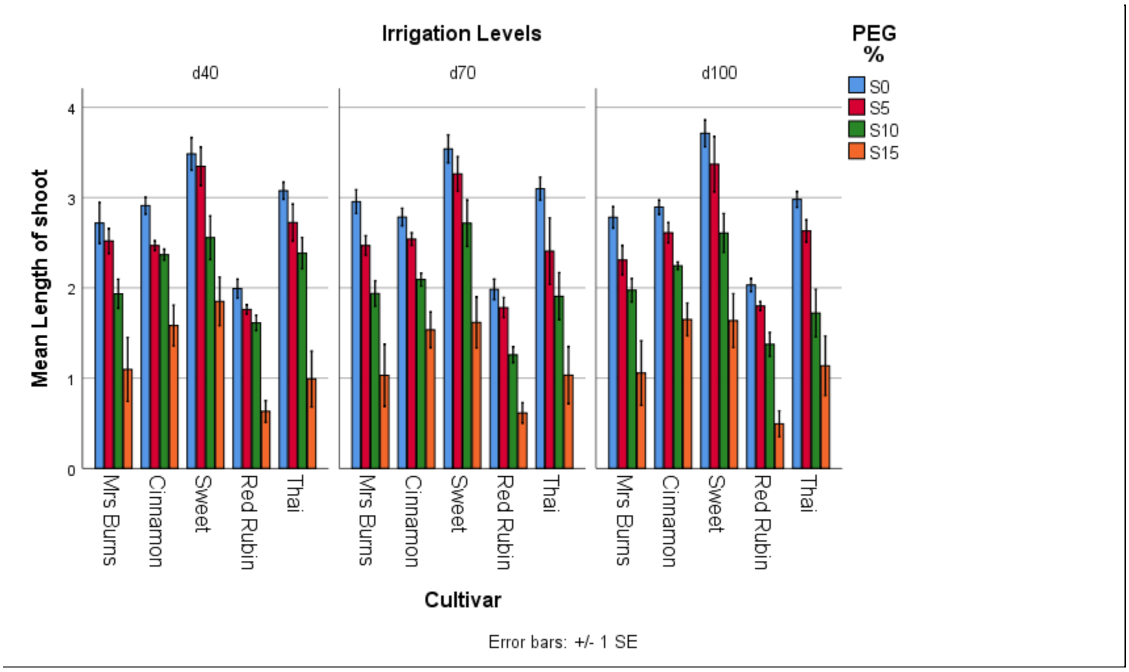

Figure S5. Shoot length of the seedling of the five basil cultivars ("Mrs Burns", "Cinnamon", "Sweet", "Red Rubin" and "Thai"), at the three levels of irrigation (d_{40}, d_{70} and d_{100}) and at the four concentrations of PEG, S_0 for 0% PEG, S_5 for 5% PEG, S_{10} for 10% PEG and S_{15} for 15% PEG. Data presented are the observed mean values averaged over the two years of experimentation. Error bars correspond to the individual standard errors of the mean values. Each mean value has been estimated utilizing n=8 measurements.

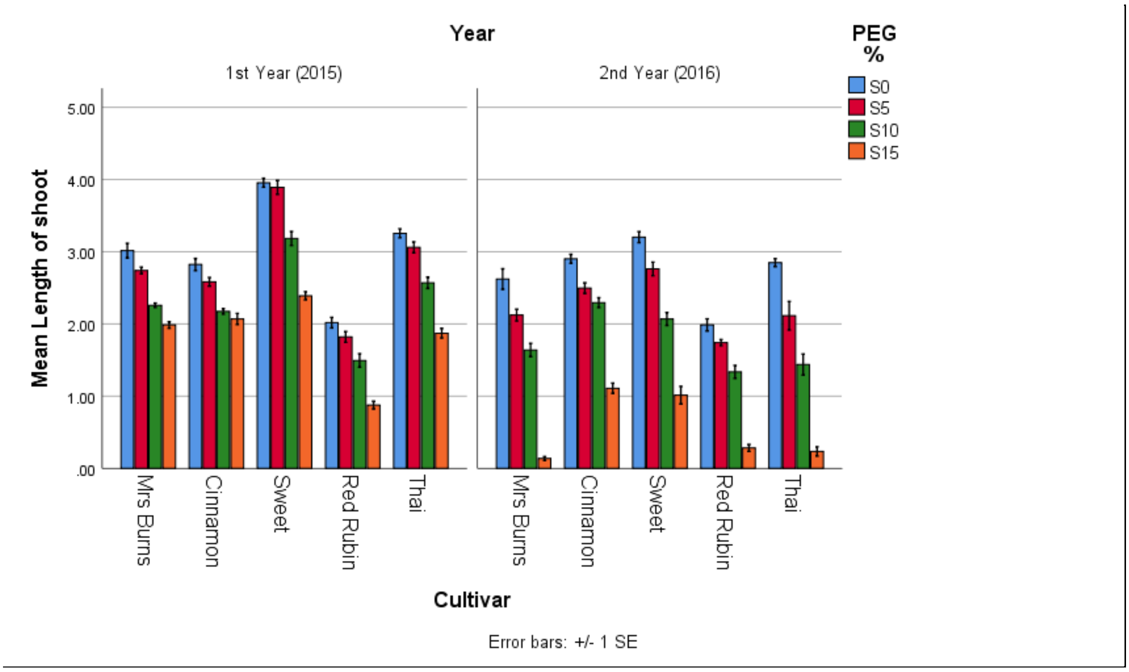

Figure S6. Shoot length of the seedling of the five basil cultivars ("Mrs Burns", "Cinnamon", "Sweet", "Red Rubin" and "Thai"), at the four concentrations of PEG, S_0 for 0% PEG, S_5 for 5% PEG, S_{10} for 10% PEG and S_{15} for 15% PEG, at the two years of experimentation. Data presented are the observed mean values averaged over the three irrigation levels. Error bars correspond to the individual standard errors of the mean values. Each mean value has been estimated utilizing n=12 measurements.

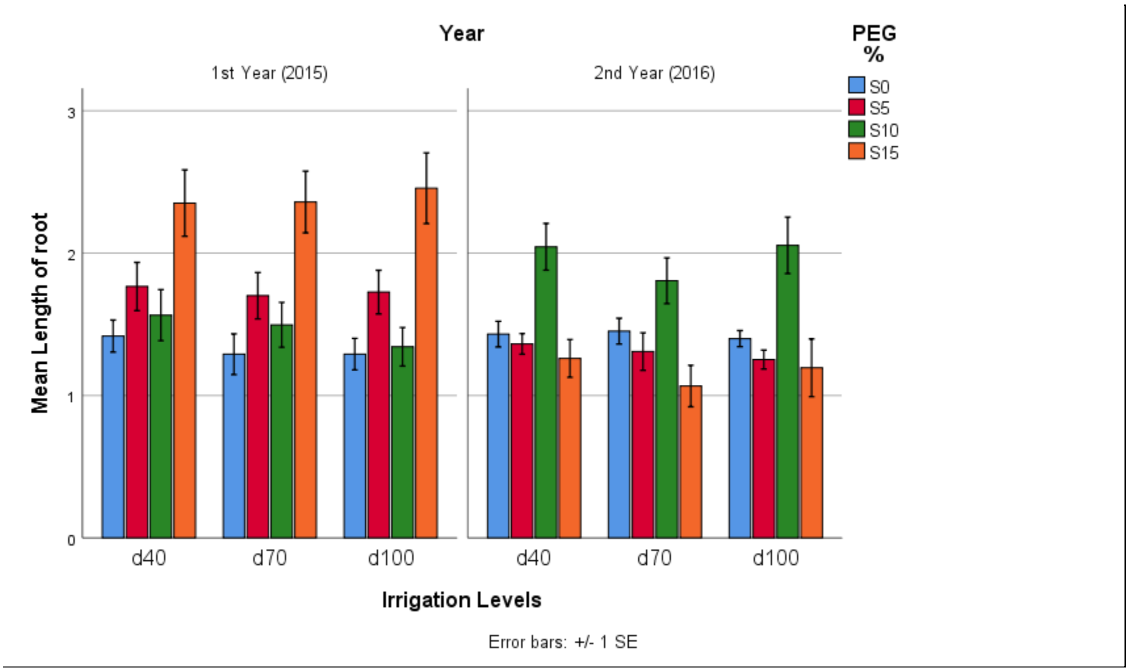

Figure S7. Root length at the three levels of irrigation (d_{40}, d_{70} and d_{100}), at the four PEG concentrations (S_0, S_5, S_{10} and S_{15}), at the two years of experimentation. Data presented are the observed mean values averaged over the five cultivars. Error bars correspond to the individual standard errors of the mean values. Each mean value has been estimated utilizing n=20 measurements.

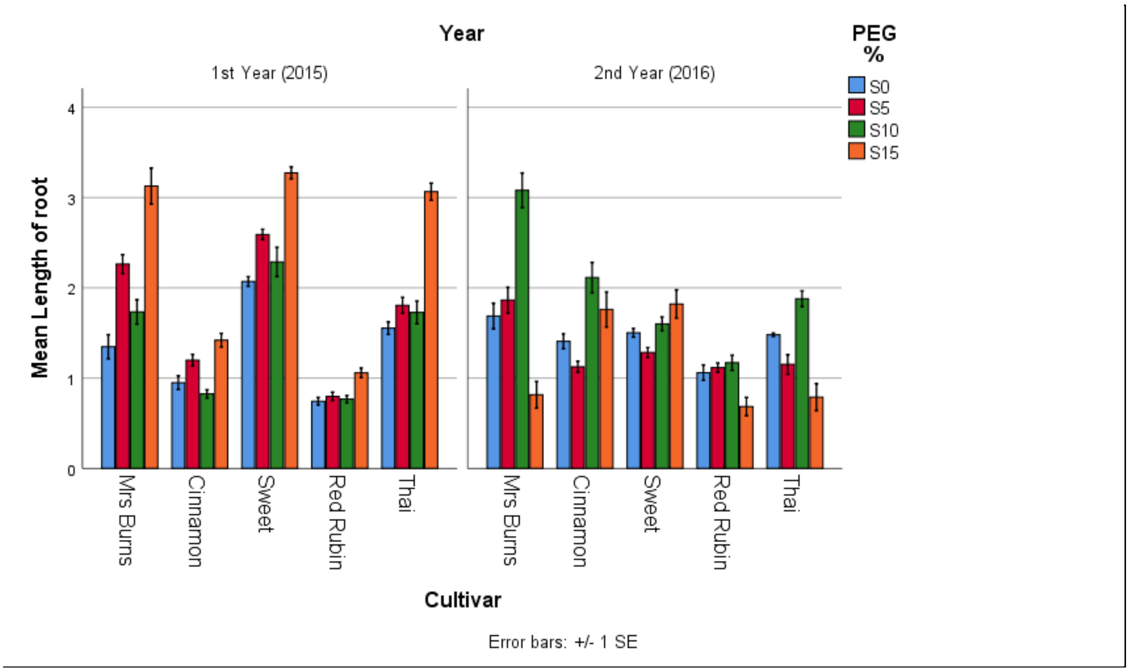

Figure S8. Root length of the five basil cultivars ("Mrs Burns", "Cinnamon", "Sweet", "Red Rubin" and "Thai"), at the four PEG concentrations (S_0, S_5, S_{10} and S_{15}), at the two years of experimentation. Data presented are the observed mean values averaged over the three irrigation levels. Error bars correspond to the individual standard errors of the mean values. Each mean value has been estimated utilizing n=12 measurements.

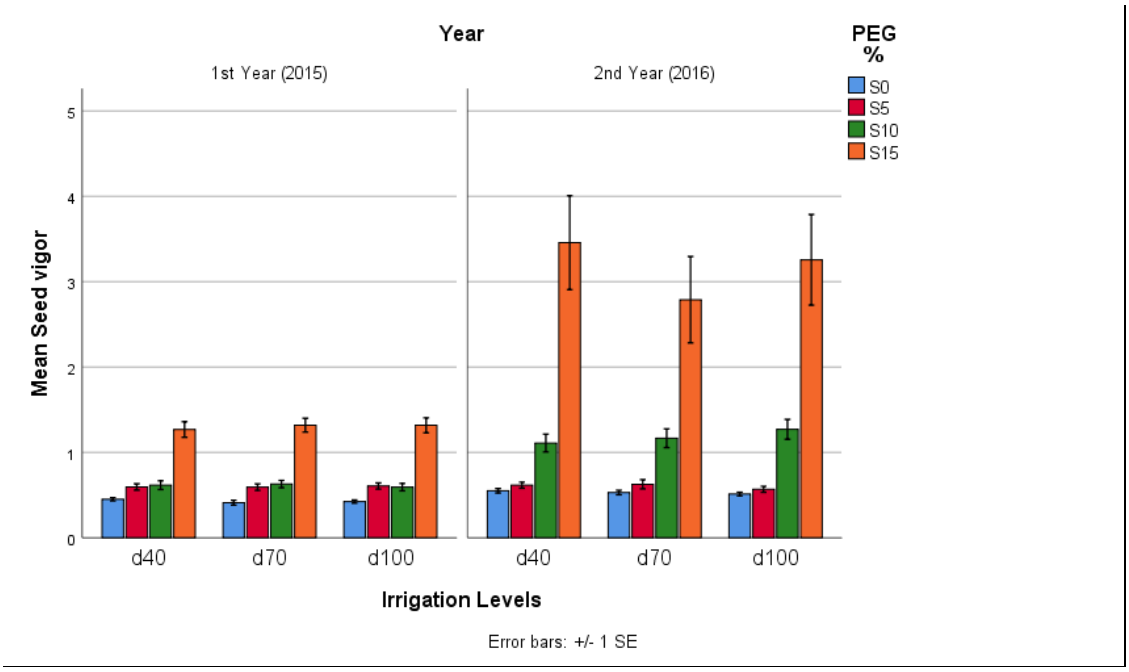

Figure S9. Seed vigor at the three levels of irrigation (d_{40}, d_{70}, and d_{100}), at the four PEG concentrations (S_0, S_5, S_{10} and d_{15}), at the two years of experimentation. Data presented are the observed mean values averaged over the five cultivars. Error bars correspond to the individual standard errors of the mean values. Each mean value has been estimated utilizing *n*=20 measurements.

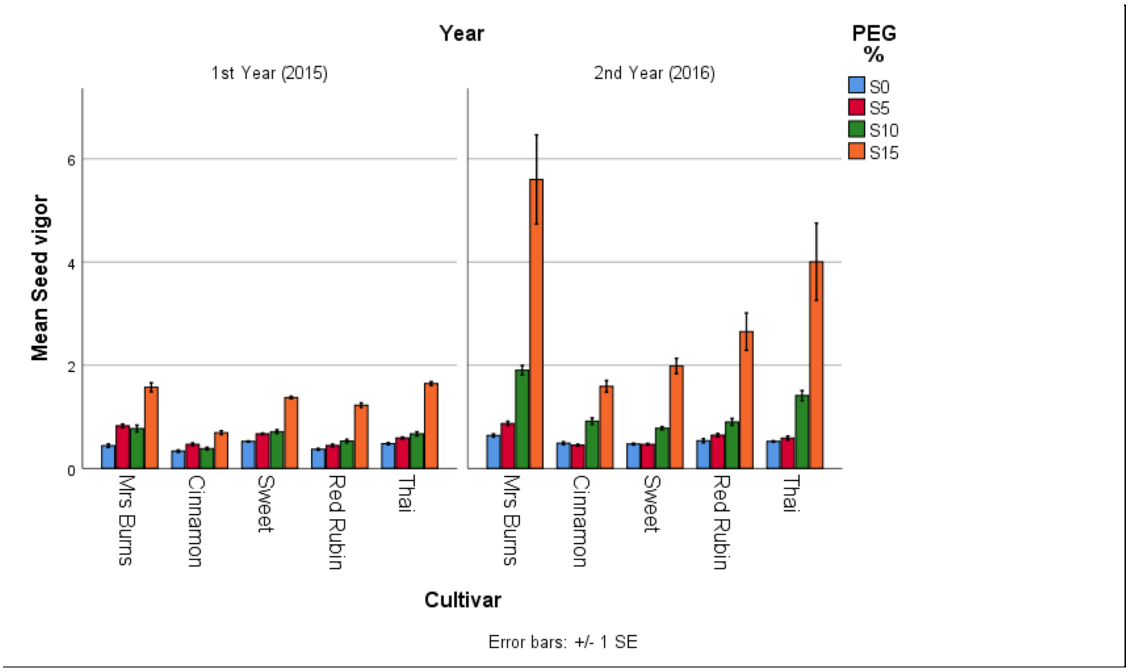

Figure S10. Seed vigor of the five basil cultivars (“Mrs Burns”, “Cinnamon”, “Sweet”, “Red Rubin”, and “Thai”), at the four PEG concentrations (S_0, S_5, S_{10}, and S_{15}) at the two years of experimentation. Data presented are the observed mean values averaged over the three irrigation levels. Error bars correspond to the individual standard errors of the mean values. Each mean value has been estimated utilizing n=12 measurements.

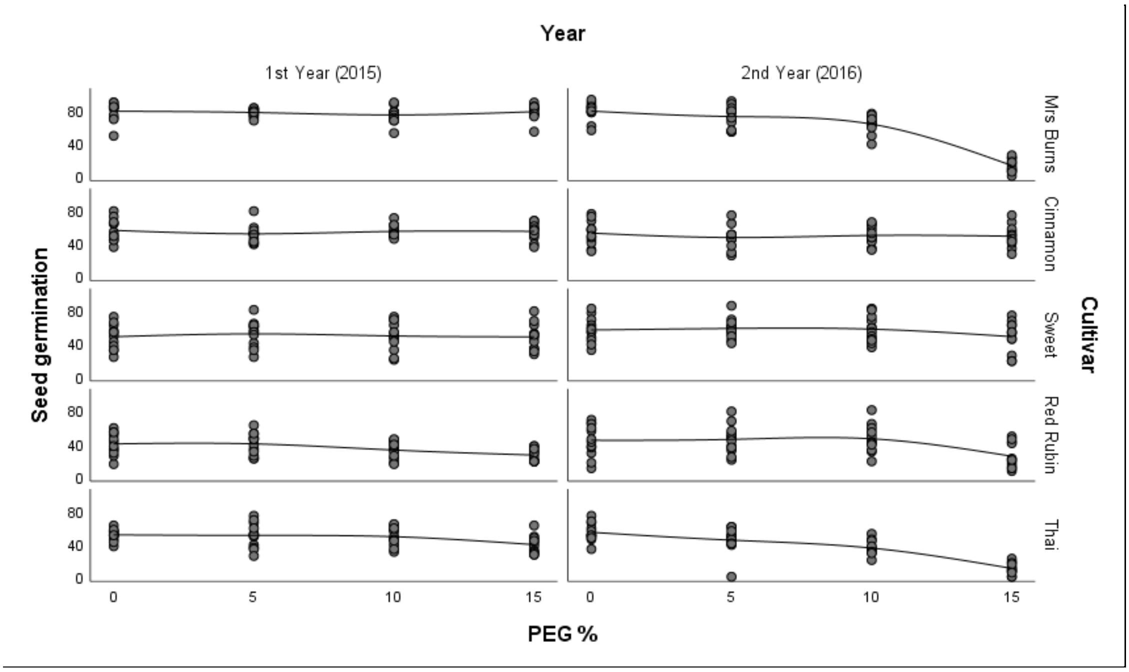

Figure S4.1. Variability and trend, relative to PEG concentrations, of seed germination, over the three irrigation levels, of the five basil cultivars ("Mrs Burns", "Cinnamon", "Sweet", "Red Rubin" and "Thai"), at the two years of experimentation.

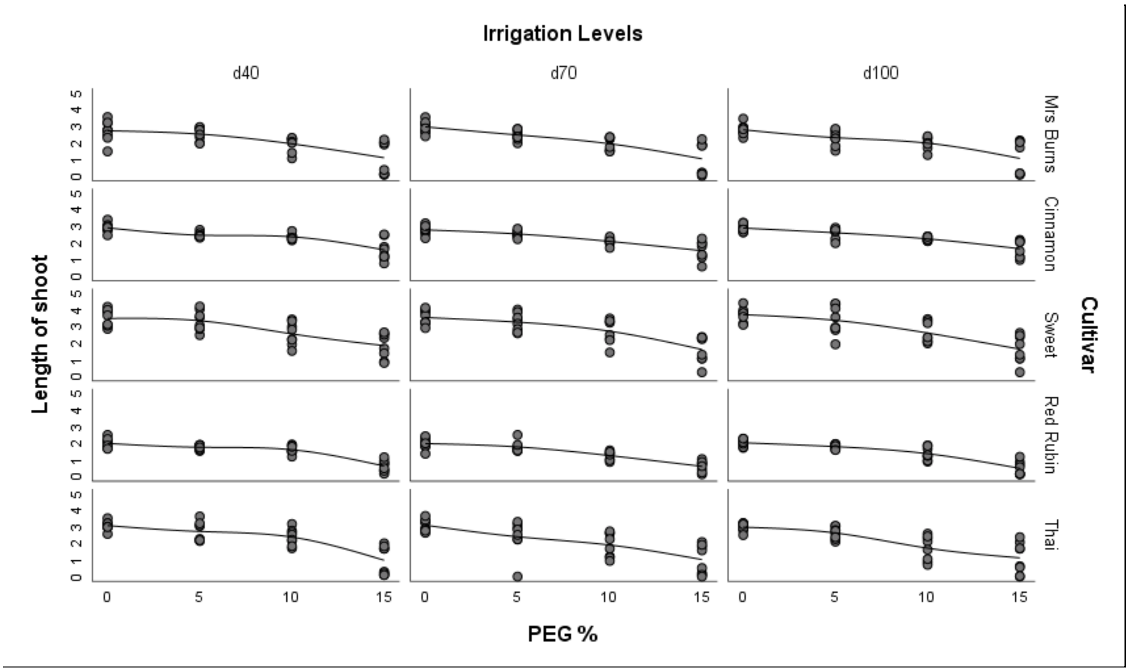

Figure S5.1. Variability and trend, relative to PEG concentrations, of shoot length of the seedling, over the two years of experimentation, of the five basil cultivars (“Mrs Burns”, “Cinnamon”, “Sweet”, “Red Rubin” and “Thai”), at the three levels of irrigation (d_{40}, d_{70} and d_{100}).

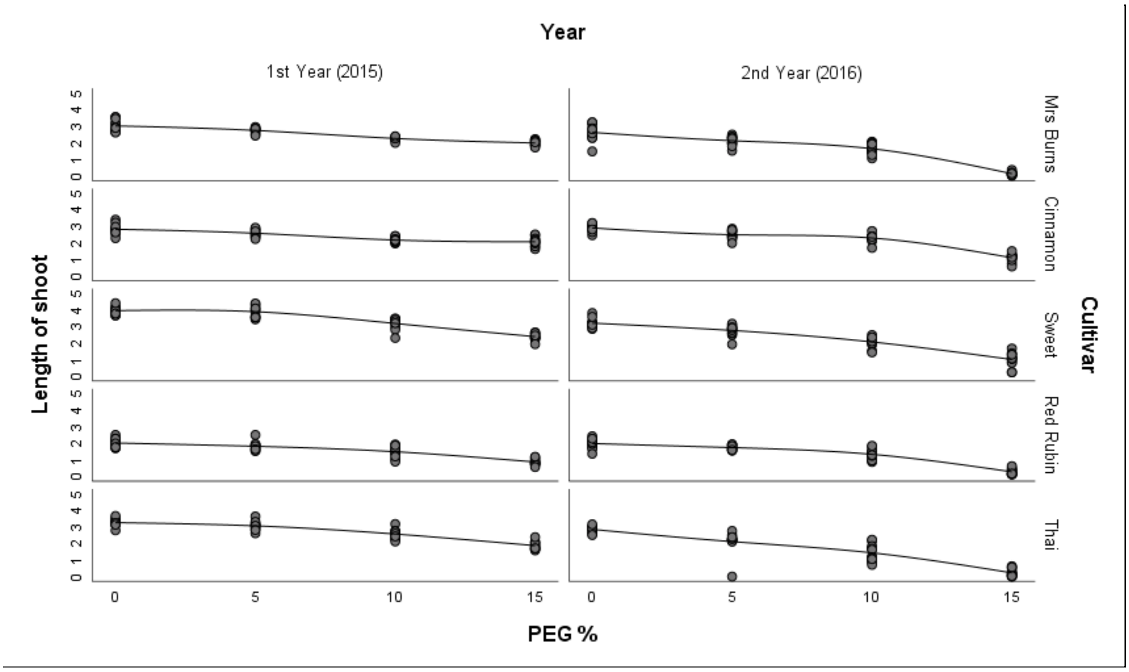

Figure S6.1. Variability and trend, relative to PEG concentrations, of shoot length of the seedling, over the three irrigation levels, of the five basil cultivars ("Mrs Burns", "Cinnamon", "Sweet", "Red Rubin" and "Thai"), at the two years of experimentation.

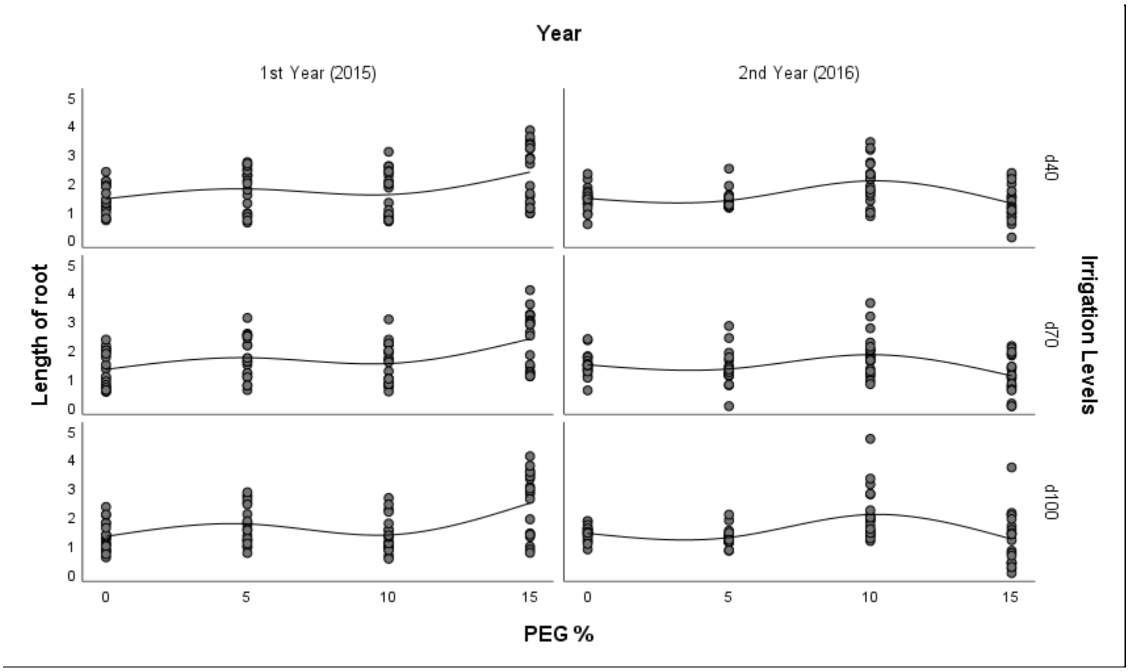

Figure S7.1. Variability and trend, relative to PEG concentrations, of root length, over the five cultivars, at the three levels of irrigation (d_{40}, d_{70} and d_{100}), at the two years of experimentation.

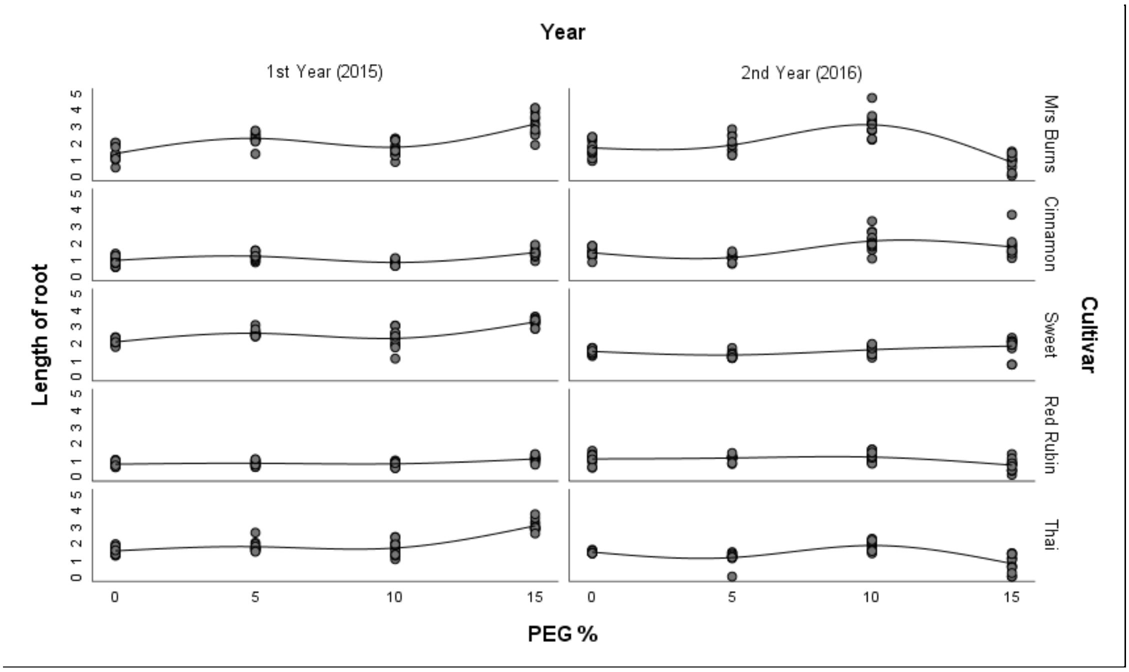

Figure S8.1.Variability and trend, relative to PEG concentrations, of root length, over the three irrigation levels, of the five basil cultivars ("Mrs Burns", "Cinnamon", "Sweet", "Red Rubin" and "Thai"), at the two years of experimentation.

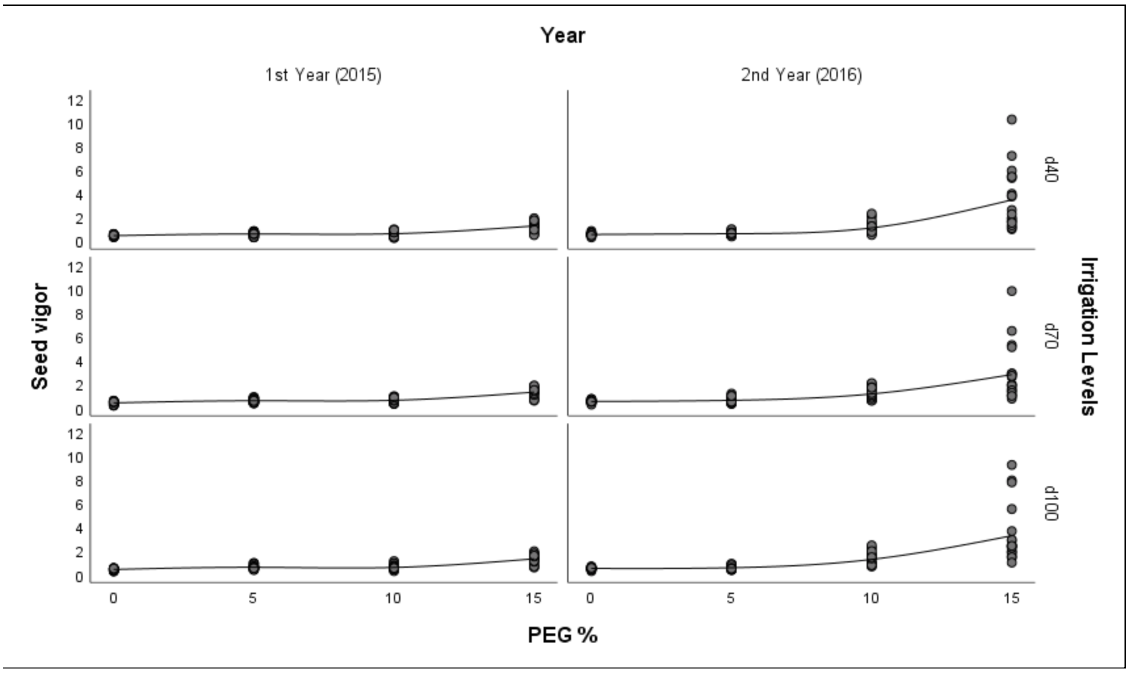

Figure S9.1. Variability and trend, relative to PEG concentrations, of seed vigor, over the five cultivars, at the three levels of irrigation (d_{40}, d_{70}, and d_{100}), at the two years of experimentation.

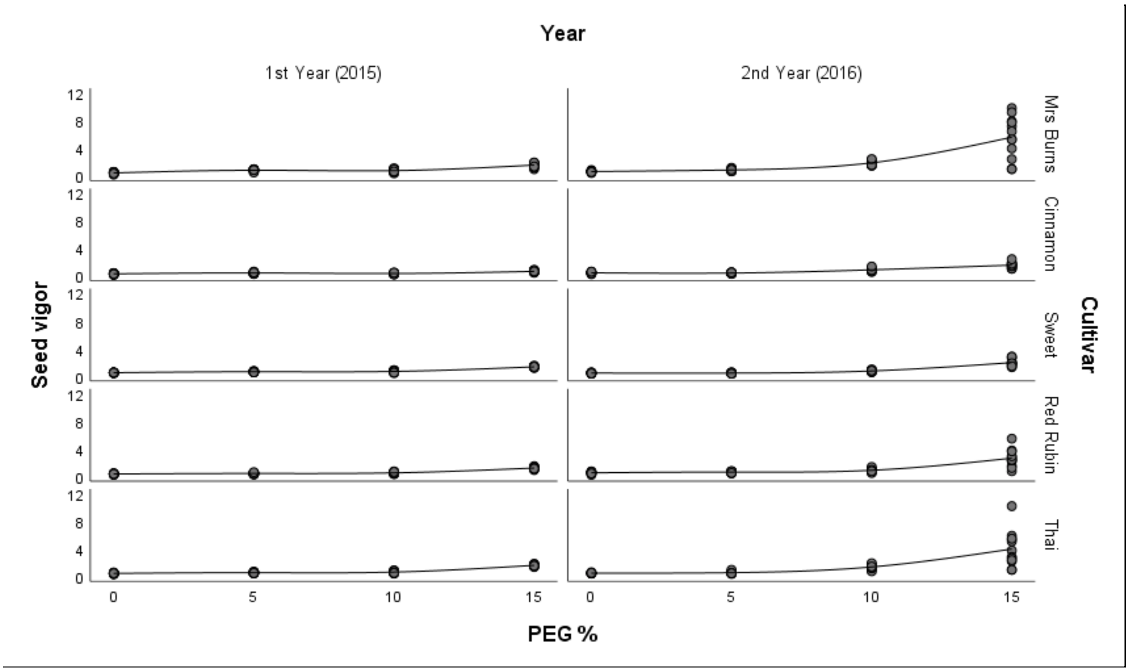

Figure S10.1. Variability and trend, relative to PEG concentrations, of seed vigor, over the three irrigations levels, of the five basil cultivars (“Mrs Burns”, “Cinnamon”, “Sweet”, “Red Rubin”, and “Thai”), at the two years of experimentation

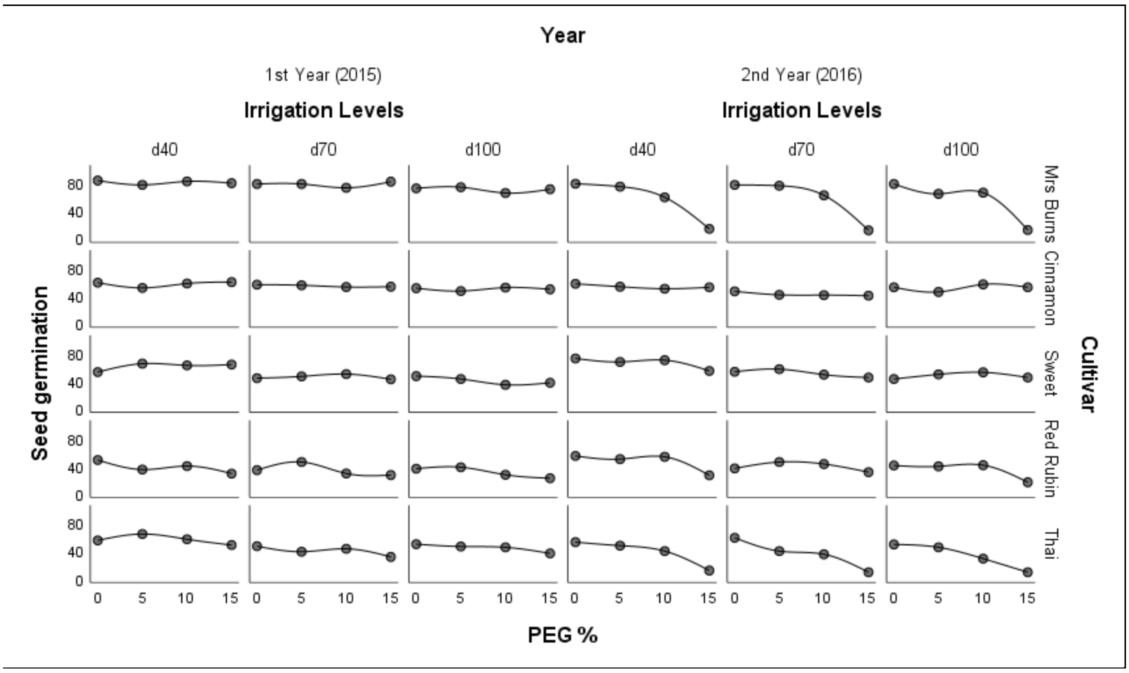

Figure S11. Trend of seed germination relative to PEG concentrations in all factors' levels combinations. Plotted data are the mean values of n=4 measurements.

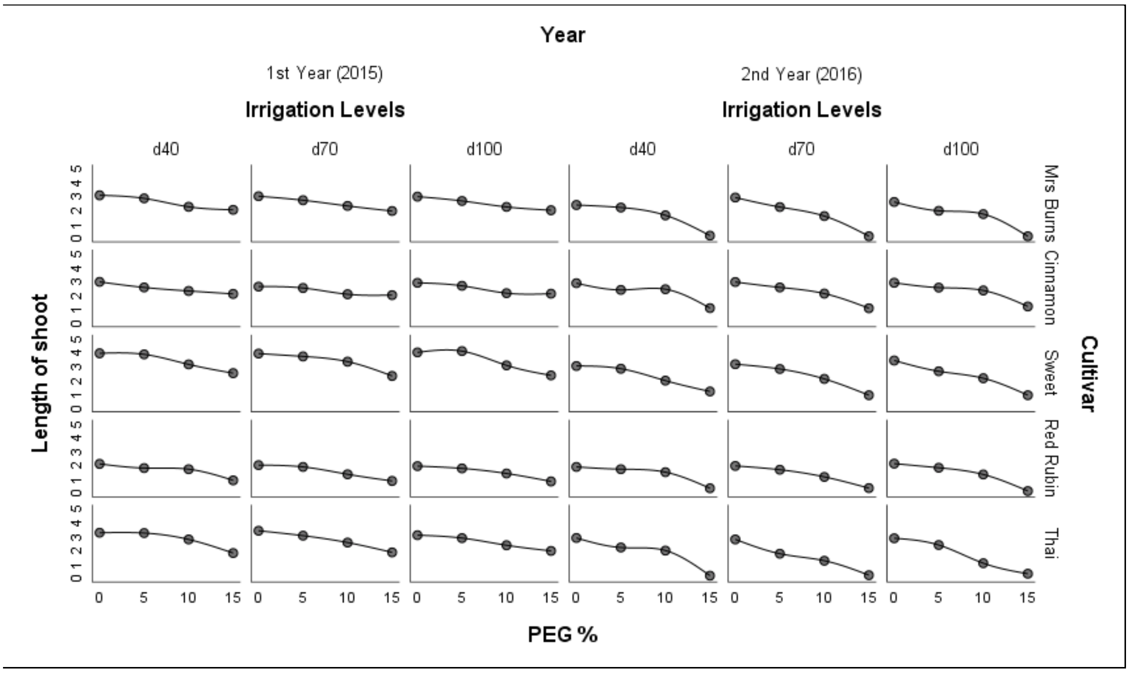

Figure S12. Trend of shoot length relative to PEG concentrations in all factors' levels combinations. Plotted data are the mean values of n=4 measurements.

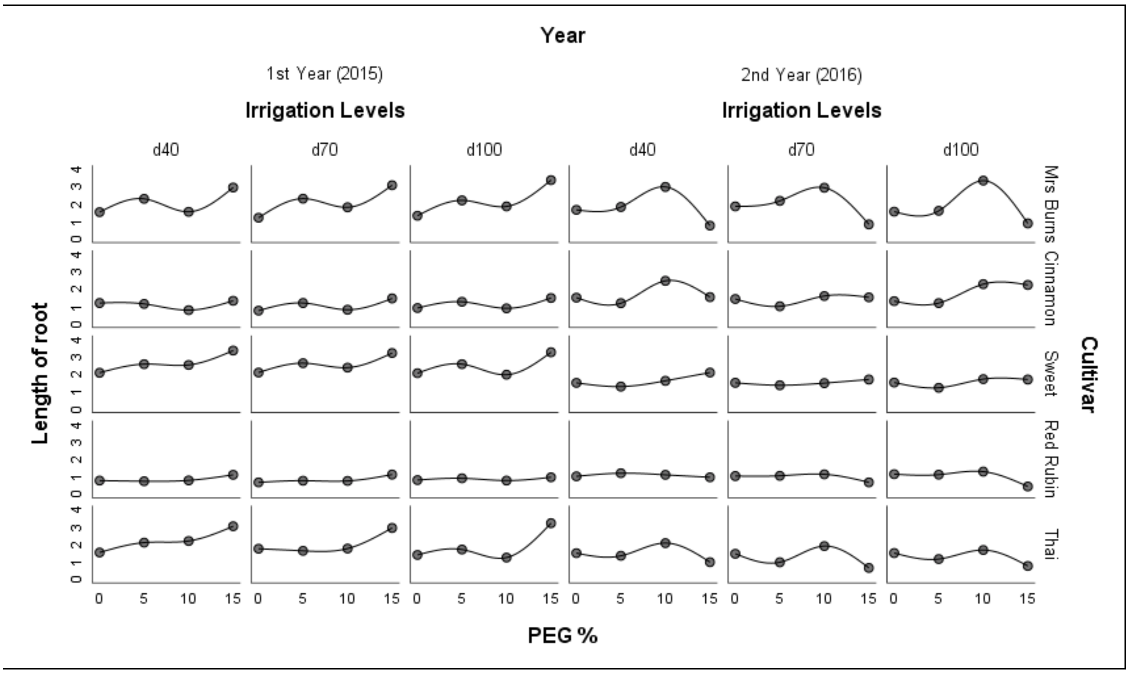

Figure S13. Trend of root length relative to PEG concentrations in all factors' levels combinations. Plotted data are the mean values of *n*=4 measurements.

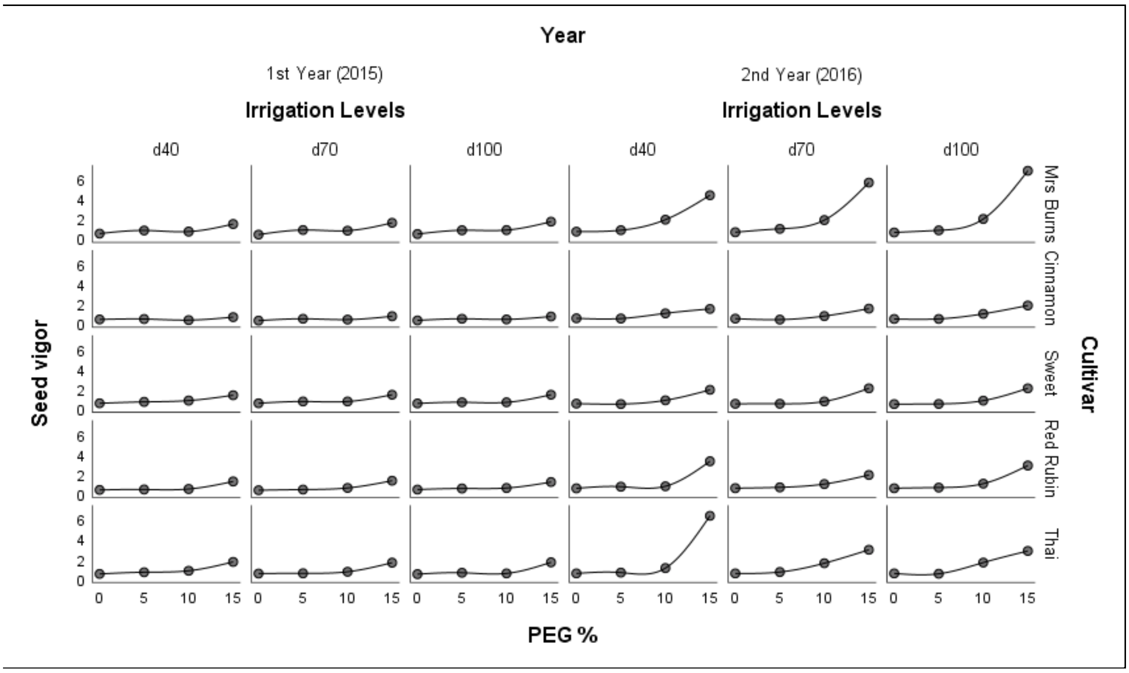

Figure S14. Trend of seed vigor relative to PEG concentrations in all factors' levels combinations. Plotted data are the mean values of *n*=4 measurements.
