# Supplementary material for: Effect of Low Water Availability on Seed Yield and Seed Quality of Basil (Ocimum basilicum)
Source: Plants (Basel). 2023 Mar 1;12(5):1094. doi: 10.3390/plants12051094 (PMC10005375; doi:10.3390/plants12051094)
Supplement: Supplementary file 1 [file plants-12-01094-s001.zip › Supplementary tables.pdf]

Supplementary material: Tables

Table S1. Main effect (mean ± common SE and median values) of the two years of experimentation on seed yield and thousand seed weight.

Year		Seed Yield (gm^{-2})	Thousand seed weight (g)
2015	Mean	116.08±5.13	1.00±0.033
	Median	91.00	0.99
2016	Mean	105.42±5.13	1.04±0.033
	Median	76.00	1.12
Total	Mean	110.75±3.63	1.02±0.023
	Median	82.00	1.06

Table S2. Main effect (mean ± common SE and median values) of the three irrigation treatments on seed yield and thousand seed weight.

Irrigation		Seed Yield (gm^{-2})	Thousand seed weight (g)
d40	Mean	96.35±6.29	0.99±0.040
	Median	76.00	1.06
d70	Mean	105.30±6.29	1.02±0.040
	Median	83.50	0.99
d100	Mean	130.60±6.29	1.05±0.040
	Median	89.00	1.08

Table S3. Main effect (mean ± common SE and median values) of the basil cultivars on seed yield and thousand seed weight.

Cultivar		Seed Yield (gm^{-2})	Thousand seed weight (g)
Mrs Burns	Mean	184.92±8.12	1.20±0.05
	Median	188.50	1.24
Cinnamon	Mean	172.38±8.12	1.09±0.05
	Median	183.00	1.14
Sweet	Mean	64.17±8.12	1.14±0.05
	Median	63.50	1.10
Red Rubin	Mean	59.38±8.12	.80±0.05
	Median	59.00	.86
Thai	Mean	72.92±8.12	.88±0.05
	Median	66.00	.83

Table S4. Main effect (mean ± common SE and median values) of the two years of experimentation on seed germination, length of shoot, length of root, and seed vigor.

Year		Seed germination (%)	Length of shoot (cm)	Length of root (cm)	Seed vigor
2015	Mean	51.86±0.62	2.50±0.02	1.73±0.02	0.73±0.042
	Median	50.00	2.43	1.56	0.59
2016	Mean	46.90±0.62	1.82±0.02	1.47±0.02	1.37±0.042
	Median	48.33	1.99	1.40	0.78
Total	Mean	49.38±0.44	2.16±0.01	1.60±0.015	1.05±0.03
	Median	50.00	2.25	1.43	0.66

Table S5. Main effect (mean ± common SE and median values) of the three irrigation treatments on seed germination, length of shoot, length of root and seed vigor.

Irrigation		Seed germination (%)	Length of shoot (cm)	Length of root (cm)	Seed vigor
d_{40}	Mean	55.06±0.75	2.20±0.022	1.65±0.03	1.08±0.05
	Median	55.84	2.26	1.49	0.68
d_{70}	Mean	47.47±0.75	2.13±0.022	1.56±0.03	1.01±0.05
	Median	46.67	2.26	1.45	0.68
d_{100}	Mean	45.60±0.75	2.15±0.022	1.59±0.03	1.07±0.05
	Median	45.84	2.21	1.39	0.63

Table S6. Main effect (mean ± common SE and median values) of the five basil cultivars on seed germination, length of shoot, length of root and seed vigor.

Cultivar		Seed germination (%)	Length of shoot (cm)	Length of root (cm)	Seed vigor
Mrs Burns	Mean	66.20±0.97	2.06±0.03	1.99±0.034	1.58±0.066
	Median	74.17	2.25	2.00	0.90
Cinnamon	Mean	51.16±0.97	2.31±0.03	1.35±0.034	0.66±0.066
	Median	50.00	2.39	1.25	0.50
Sweet	Mean	51.61±0.97	2.81±0.03	2.05±0.034	0.87±0.066
	Median	53.33	2.92	1.98	0.67
Red Rubin	Mean	36.81±0.97	1.44±0.03	0.92±0.034	0.91±0.066
	Median	36.67	1.60	0.92	0.63
Thai	Mean	41.11±0.97	2.17±0.03	1.68±0.034	1.24±0.066
	Median	45.00	2.38	1.55	0.64

Table S7. Main effect (mean ± common SE and median values) of the four PEG concentrations on seed germination, length of shoot, length of root and seed vigor.

PEG %		Seed germination (%)	Length of shoot (cm)	Length of root (cm)	Seed vigor
S0	Mean	55.32±0.87	2.86±0.025	1.38±0.03	0.48±0.06
	Median	53.33	2.91	1.39	0.49
S5	Mean	53.19±0.87	2.53±0.025	1.52±0.03	0.60±0.06
	Median	51.67	2.50	1.31	0.57
S10	Mean	50.41±0.87	2.05±0.025	1.72±0.03	0.90±0.06
	Median	48.33	2.13	1.68	0.78
S15	Mean	38.60±0.87	1.20±0.025	1.78±0.03	2.23±0.06
	Median	37.50	1.15	1.46	1.57
